# Racial and socioeconomic disparities in multimorbidity and associated healthcare utilisation and outcomes in Brazil: a cross-sectional analysis of three million individuals

**DOI:** 10.1186/s12889-021-11328-0

**Published:** 2021-07-01

**Authors:** Thomas Hone, Jonathan Stokes, Anete Trajman, Valeria Saraceni, Claudia Medina Coeli, Davide Rasella, Betina Durovni, Christopher Millett

**Affiliations:** 1 Public Health Policy Evaluation Unit, Imperial College London, Charing Cross Hospital, St Dunstan’s Road, London, W6 8R UK; 2 Health Organisation, Policy, and Economics, Centre for Primary Care and Health Services Research, University of Manchester, Manchester, UK; 3 Programa de Pós-graduação em Clínica Médica and Mestrado Profissional em Atenção Primária à Saúde, Federal University of Rio de Janeiro, Rio de Janeiro, Brazil; 4 Secretaria Municipal de Saúde do Rio de Janeiro, Rio de Janeiro, Brazil; 5 Instituto de Estudos em Saúde Coletiva, Universidade Federal do Rio de Janeiro, Rio de Janeiro, Brazil; 6 Instituto de Saúde Coletiva, Universidade Federal da Bahia, Salvador, Brazil; 7 Secretaria Municipal de Saúde do Rio de Janeiro, Rio de Janeiro, Brazil; 8 Center of Data and Knowledge Integration for Health (CIDACS), Instituto Gonçalo Muniz, Fundação Oswaldo Cruz, Salvador, Brazil

**Keywords:** Multimorbidity, Chronic conditions, Mortality, Utilisation, Hospitalisations, Middle-income country, Brazil

## Abstract

**Background:**

Evidence is limited on racial/ethnic group disparities in multimorbidity and associated health outcomes in low- and middle-income countries hampering effective policies and clinical interventions to address health inequalities.

**Methods:**

This study assessed race/ethnic and socioeconomic disparities in the prevalence of multimorbidity and associated healthcare utilisation, costs and death in Rio de Janeiro, Brazil. A cross-sectional analysis was carried out of 3,027,335 individuals registered with primary healthcare (PHC) services. Records included linked data to hospitalisation, mortality, and welfare-claimant (Bolsa Família) records between 1 Jan 2012 and 31 Dec 2016. Logistic and Poisson regression models were carried out to assess the likelihood of multimorbidity (two or more diagnoses out of 53 chronic conditions), PHC use, hospital admissions and mortality from any cause. Interactions were used to assess disparities.

**Results:**

In total 13,509,633 healthcare visits were analysed identifying 389,829 multimorbid individuals (13%). In adjusted regression models, multimorbidity was associated with lower education (Adjusted Odds Ratio (AOR): 1.26; 95%CI: 1.23,1.29; compared to higher education), Bolsa Família receipt (AOR: 1.14; 95%CI: 1.13,1.15; compared to non-recipients); and black race/ethnicity (AOR: 1.05; 95%CI: 1.03,1.06; compared to white). Multimorbidity was associated with more hospitalisations (Adjusted Rate Ratio (ARR): 2.75; 95%CI: 2.69,2.81), more PHC visits (ARR: 3.46; 95%CI: 3.44,3.47), and higher likelihood of death (AOR: 1.33; 95%CI: 1.29,1.36). These associations were greater for multimorbid individuals with lower educational attainment (five year probability of death 1.67% (95%CI: 1.61,1.74%) compared to 1.13% (95%CI: 1.02,1.23%) for higher education), individuals of black race/ethnicity (1.48% (95%CI: 1.41,1.55%) compared to 1.35% (95%CI: 1.31,1.40%) for white) and individuals in receipt of welfare (1.89% (95%CI: 1.77,2.00%) compared to 1.35% (95%CI: 1.31,1.38%) for non-recipients).

**Conclusions:**

The prevalence of multimorbidity and associated hospital admissions and mortality are greater in individuals with black race/ethnicity and other deprived socioeconomic groups in Rio de Janeiro. Interventions to better prevent and manage multimorbidity and underlying disparities in low- and middle-income country settings are needed.

**Supplementary Information:**

The online version contains supplementary material available at 10.1186/s12889-021-11328-0.

## Background

Noncommunicable diseases (NCDs) remain the leading cause of death and disability worldwide. However, 77% of NCD deaths and 82% of NCD DALYs lost globally are in low- and middle-income countries (LMICs) [1]. The estimated economic burden of leading NCDs between 2011 and 2030 is USD $47 trillion (2010 USD $) with nearly half (USD $21 trillion) in LMICs [2]. Appropriate prevention and management of risk factors and chronic conditions is essential to address the NCD burden [3].

Multimorbidity (two or more chronic conditions) strains health systems attempting to manage the growing burden of NCDs. Multimorbid individuals report worse quality of life [4] and increased functional decline [5], incur higher healthcare costs [5], and are at increased risk of death [6]. Individuals with deprived socioeconomic status and low educational attainment have a higher prevalence of multimorbidity [7–13], including in LMICs [14–16]. Accumulation of risk factors, chronic stress, and poorer healthcare access in deprived socioeconomic groups drive these disparities. Multimorbidity onset can be up to 15 years earlier in deprived populations compared to affluent populations [17].

Research on multimorbidity in LMICs is extremely limited [18, 19] – particularly examining racial/ethnic and socioeconomic group disparities. Studies in high-income countries show higher rates of multimorbidity among Hispanics and African Americans in the USA [8–11], South-Asian and black individuals in the UK [12], and ethnic minorities in the Netherlands [13]. Similarly, evidence from the USA [11] and cross-country studies in LMICs [14–16] show those with lower education have higher rates of multimorbidity. Studies from LMICs almost exclusively rely on household surveys which are subject to recall bias and are often underpowered to detect disparities.

Brazil is an important setting for evaluating multimorbidity. It is a large middle-income country that has expanded universal health services in the context of stark disparities in health outcomes between socioeconomic and race/ethnic groups [20]. Brazil’s GINI index of income inequality was 53 in 2019 - one of the most unequal in the world. More than half of Brazilians aged 25 or older have not completed secondary school [21]. Nearly a third (32.9%) of black or pardo Brazilians earn less than US$5.50 a day (compared to 15.4% of white Brazilians), whilst 9.1% of black/pardo Brazilians are illiterate compared to 3.9% of white Brazilians [22]. Primary health care (PHC), under the Family health Strategy, have been expanded nationally since the mid 1990s covering around 60% of the population in 2018 [23]. PHC services are publicly-funded and free at the point of care, and include comprehensive preventative and acute care provided by multidisciplinary teams [24]. With a sizeable private sector, PHC in Brazil generally covers low and middle-income populations. However, there are major health system fragilities [23] and growing NCD risk factors [25]. Nationally, an estimated 42 million individuals (22–23% of adults) are multimorbid [26, 27], rising to 68% for those over 50 years of age [28], with higher rates of multimorbidity for those with lower education [26–28]. Brazilian studies utilising medical records with statistical power to examine disparities in multimorbidity prevalence and outcomes are scarce. This study uses a large dataset of three million individuals of all ages over the life course registered with PHC services in the city of Rio de Janeiro. It firstly assesses the prevalence of multimorbidity and associated risk factors, and secondly explores the association of multimorbidity with healthcare utilisation, patients’ healthcare costs, and death between race/ethnicity and socioeconomic groups.

## Methods

### Study design

A cross-sectional analysis of PHC registered individuals with linked welfare, PHC, hospitalisation, and mortality records.

### Data sources

PHC registration records of 3,027,335 individuals of all ages in Rio de Janeiro city were obtained - covering 47% of the city population. The study population is low-income as PHC services are focused in poorer areas. All individuals who registered with PHC up to 31 Dec 2016 were included. Records were linked to welfare claimant records (*Cadastro Unico*), PHC electronic medical records, hospitalisation admission records (Sistema de Informações Hospitalares; SIH), and mortality records (Sistema de Informações sobre Mortalidade; SIM) - all covering the five-year period from 1 Jan 2012 to 31 Dec 2016. Datasets were obtained from the Secretariat for Health in Rio de Janeiro and linked using tax numbers, date of birth, and phonetic matching of names. The linkage methods involved deterministic and probabilistic processes with manual review. Probabilistic matching varied from 8 to 15%, depending on the databases being linked. Details on the linkage are published elsewhere [29, 30].

From PHC registration records, individuals’ characteristics were collated including age, sex, race/ethnicity, highest educational attainment, and private insurance usage. Welfare claimant records identified individuals in receipt of welfare (conditional cash transfers; *Bolsa Família*) and monthly expenditures on medicines. From PHC consultations, hospitalisation admission records, and mortality records, ICD-10 codes and PHC procedure codes were obtained. There were 3,173,289 individuals in the original dataset (registered up to 31 Dec 2016), of which 145,954 (4.6%) were removed - 76,418 due to missing data on sex, race, date of birth or date of death; 67,170 duplicates; and 2366 records with deaths before 1 Jan 2012.

To assess multimorbidity, 53 chronic conditions were examined (Additional File 1 for conditions and ICD-10/procedure codes based on previous studies [17, 19, 31, 32]). Individuals were assigned chronic disease diagnoses based on criteria relating to timing of diagnoses (e.g. diagnoses within last 2 years), although for most conditions this was if any relevant diagnosis was ever recorded. Multimorbidity was defined as having two or more chronic disease diagnoses.

The resulting dataset contained PHC registered individuals and their chronic disease diagnoses, demographic and socioeconomic characteristics, counts of PHC consultations (all consultations including treatment and preventative care) and hospital admissions (emergency and elective) and mortality outcomes. Hospital admissions due to childbirth were excluded (ICD10 O00-O99.9, Z32-Z39.9). For a subset of the population (welfare claimants), household monthly expenditure on medicines was available. This was the reported monthly household expenditure on medicines (R$s). Demographic and socioeconomic characteristics were encoded as: sex (male; female); 5 year age groups; self-reported race/ethnicity (white; black; pardo (mixed race); Asian (*Amarelo*); or indigenous); the individual’s highest educational attainment (none, pre-school or literacy class; elementary School (Grades 1–4); elementary School (Grades 5+); high-school; higher education; or missing); if individual was in a Bolsa Família recipient household (yes; no); and if individual had private insurance (yes; no).

### Statistical analysis

#### Prevalence of multimorbidity and associated risk factors

The prevalence of multimorbidity across race/ethnicity and socioeconomic groups was reported. The most common chronic conditions and those chronic conditions that contributed most to hospitalisations and deaths were reported. Logistic regression was employed to explore risk of multimorbidity by race/ethnicity and socioeconomic factors. Covariates were sex, age, race, educational group, Bolsa Família receipt, private insurance, and if individuals had any PHC use or hospitalisation. Adjusted odds ratios (AOR) were reported. Due to the high level of missing data on education (26.9%), analyses were conducted including the missing category for comparison.

#### Associations between multimorbidity, healthcare use, expenditures and death, and disparities across race/ethnic and socioeconomic groups

Regression modelling examined association between multimorbidity and PHC and hospital usage, health expenditures and mortality. Models were adjusted for sex, age, race/ethnicity, educational group, Bolsa Família receipt, and private insurance. Logistic regression was employed for death (binary outcome), Poisson models for counts (PHC consultations and hospital admissions), and linear regression for household health expenditures (subset of population with available data). AOR were reported for logistic regressions and adjusted rate ratios (ARR) reported for Poisson regression models.

The models were expanded with interactions to test whether the associations between multimorbidity and healthcare use, death and expenditures were different across race/ethnicity, educational attainment, and Bolsa Família status groups (i.e. three interactions per outcome). Post-regression probabilities (of death), rates (of PHC consultation and hospital admissions), and average household expenditures were predicted for the three socioeconomic groups and by multimorbidity status. These are interpreted relative to the five-year study period (i.e. five-year probability of death).

All analyses used robust standard errors and carried out in STATA® Statistical Software 15 (StataCorp LLC).

### Sensitivity analyses

Overall prevalence estimates of multimorbidity from all PHC registrants were compared to estimates from a subsample of those that used PHC services. Potential biases from high levels of missing education data were tested through multiple imputation where 10 imputations were made based on individuals’ recorded age, sex, education status, race/ethnicity, Bolsa Família receipt, insurance coverage, and PHC and hospital usage.

## Results

There were 3,027,335 individuals in the dataset registered with PHC (Table 1). Of these, 1,722,477 (56.9%) had at least one consultation with PHC between 1 Jan 2012 and 31 Dec 2016 (PHC users) whilst 226,255 (7.5%) had at least one hospital admission. Over half (1,693,999; 56.0%) of the population was female. By race/ethnicity, 51.5% were pardo (mixed race), 35.8% white and 12.0% were black. Over one-fifth (21.3%) were *Bolsa Família* recipients. Most (1,633,095; 54.0%) had elementary (grades 1–4 or grades 5+) or high schooling, whilst 3.0% (91,656) had higher education and 16.1% (487,860) had no formal education. The mean number of PHC consultations and hospitalisations per individual over the five-year period were 4.3 and 0.12 respectively. In total, 39,385 (1.3%) individuals died. There were 881,632 individuals (29.1%) with data on expenditures and a mean household expenditure on medicines of R$ 9.9 per month (USD $ 3.3 in 2015).

**Table 1:** Characteristics of the study population and prevalence of multimorbidity

	All (N (%))	Multimorbidity (N (%))
Sex		
Male	1,333,336 (44.0%)	128,545 (33.0%)
Female	1,693,999 (56.0%)	261,284 (67.0%)
Age group		
< 5 years	237,889 (7.9%)	2628 (0.7%)
5–9 years	158,402 (5.2%)	2221 (0.6%)
10–18 years	380,705 (12.6%)	5850 (1.5%)
19–24 years	284,701 (9.4%)	6458 (1.7%)
25–34 years	447,611 (14.8%)	18,343 (4.7%)
35–44 years	426,648 (14.1%)	39,273 (10.1%)
45–54 years	384,462 (12.7%)	71,719 (18.4%)
55–64 years	343,700 (11.4%)	102,293 (26.2%)
65–79 years	275,939 (9.1%)	107,709 (27.6%)
80+ years	87,278 (2.9%)	33,335 (8.6%)
Ethnicity/race		
White	1,083,884 (35.8%)	150,340 (38.6%)
Black	362,228 (12.0%)	57,923 (14.9%)
Asian (*Amarelo*)	18,624 (0.6%)	2049 (0.5%)
*Pardo* (mixed)	1,559,686 (51.5%)	179,135 (46.0%)
Indigenous	2913 (0.1%)	382 (0.1%)
Bolsa Família recipient family		
No	2,381,612 (78.7%)	327,010 (83.9%)
Yes	645,723 (21.3%)	62,819 (16.1%)
Education level		
None/Pre-school/Literacy class	487,860 (16.1%)	47,246 (12.1%)
Elementary School (Grades 1–4)	601,595 (19.9%)	129,992 (33.4%)
Elementary School (Grades 5+)	425,643 (14.1%)	65,956 (16.9%)
High-School	605,857 (20.0%)	107,566 (27.6%)
Higher Education	91,656 (3.0%)	18,448 (4.7%)
None reported (missing)	814,724 (26.9%)	20,621 (5.3%)
Private insurance?		
No	2,742,548 (90.6%)	367,158 (94.2%)
Yes	284,787 (9.4%)	22,671 (5.8%)
Primary care user		
No	1,304,858 (43.1%)	1718 (0.4%)
Yes	1,722,477 (56.9%)	388,111 (99.6%)
Public hospital user		
No	2,801,080 (92.5%)	315,595 (81.0%)
Yes	226,255 (7.5%)	74,234 (19.0%)
Total	3,027,335	389,829 (12.9%)

*PHC* primary healthcare

### Prevalence of multimorbidity and associated risk factors

To assess chronic condition diagnoses, 13,151,537 PHC consultations and 358,096 hospital admissions were analysed. The most common conditions were: hypertension (445,6901 individuals with diagnosis; 14.7%), diabetes mellitus (150,276; 5.0%), allergy (74,971; 2.5%), severe vision reduction (59,108; 2.0%), lipid metabolism disorders (52,991; 1.8%), and obesity (49,749; 1.6%) (Additional File 2). Over one-quarter of the population (835,151; 27.6%) had at least one chronic condition, whilst 12.9% (389,829) were multimorbid. Of multimorbid individuals, 228,153 (73.8%) had hypertension and 130,418 (33.5%) had diabetes mellitus (Additional File 3 and Additional File 4).

**Fig. 1:**
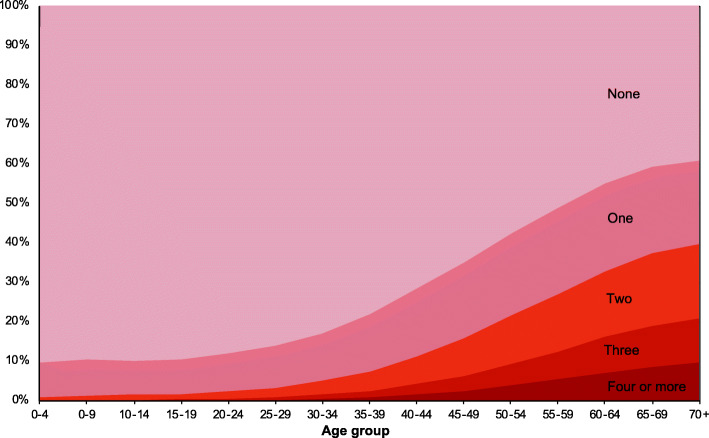
Prevalence of number of chronic conditions by age

**Fig. 2:**
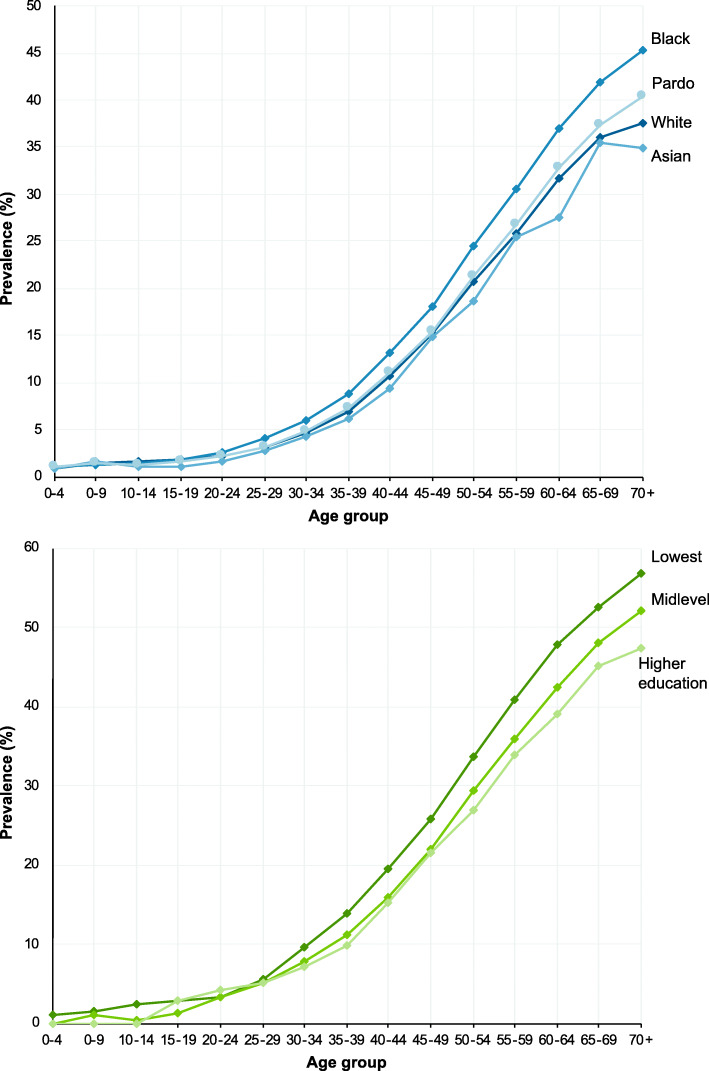
Prevalence of multimorbidity across ethnicity/racial and educational groups by age. Indigenous racial group omitted due to low numbers. Lowest education includes individuals with no schooling, literacy classes, preschool or elementary school (Grades 1–4) attainment. Midlevel education includes individuals with educational attainment of elementary School (Grades 5+) or high-school

Multimorbidity substantially increased with age (Fig. 1). Whilst on average the prevalence was 12.9%, it increased to 16.6% (379,919 individuals) for aged 18 years and over, 26.1% (337,657) for those aged 40 and over, and 37.0% (194,403) those age 60 and over (Additional File 5). There were differences by sex (15.4% of females were multimorbid; 9.6% of men) and race/ethnicity (16.0% of black, 13.9% of white, 13.1% of indigenous, 11.5% of pardo, and 11.0% of Asian). Disparities in the prevalence of multimorbidity by racial and educational groups widened with age (Fig. 2). The age at which a quarter of individuals had multimorbidity varied from 51 years for black individuals to 54 for pardo, 55 for white, and 57 for Asian (*Amarelo*).

In adjusted analyses, black individuals were 5% more likely to be multimorbid (AOR: 1.05; 95%CI: 1.03,1.06), whilst pardo individuals were 6% (AOR: 0.94; 95%CI: 0.93,0.95), Asian 11% (AOR: 0.89; 95%CI: 0.84,0.95), and indigenous individuals 19% (AOR: 0.82; 95%CI: 0.72,0.93) less likely to be multimorbid compared with white individuals (Additional File 6). The highest educated were 21% (AOR: 0.79; 95%CI: 0.77,0.81) less likely to be multimorbid than those with no education, whilst Bolsa Família recipients were 14% (AOR: 1.14; 95%CI: 1.13,1.15) more likely than non-recipients.

### Associations between multimorbidity, healthcare utilisation, expenditures, death, and disparities across race/ethnic and socioeconomic groups

Nearly half (44%; 5,727,074) of all PHC consultations, 37% (133,526) of admissions, and a third (13,172) of deaths were in multimorbid individuals. Households of multimorbid individuals spent R$ 18.1 per month on medicines compared to R$ 8.9 for non-multimorbid individuals. Adjusted logistic and Poisson regression models found multimorbid individuals had 3.5 times as many PHC consultations (ARR: 3.46; 95%CI: 3.44,3.47), 2.7 times as many hospitalisations (ARR: 2.75; 95%CI: 2.69,2.81), and were 33% more likely to die (AOR: 1.33; 95%CI: 1.29,1.36) compared to non-multimorbid individuals (Additional File 7).

**Fig. 3:**
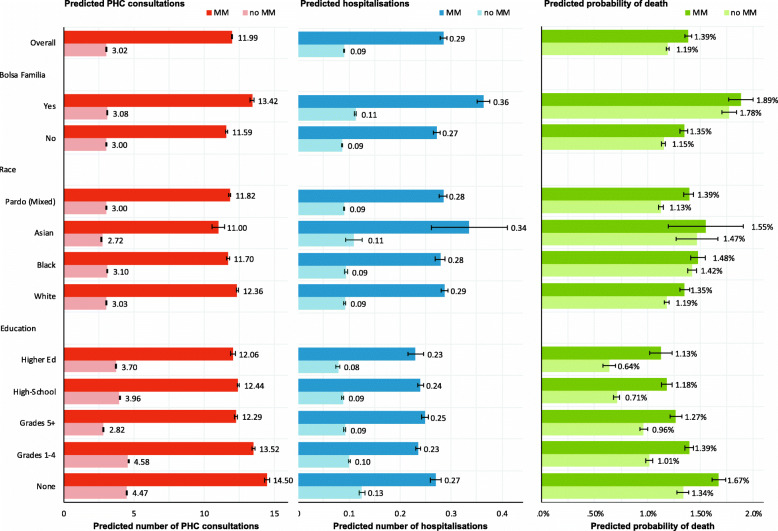
Five-year predicted numbers of PHC consultations and hospitalisations and probability of death. Predicted probabilities and rates obtained from adjusted regression models including age, sex, race, insurance status, education, Bolsa Família recipient status, multimorbidity diagnosis, and three interactions between multimorbidity status and education, race, and Bolsa Família recipient status. Predicted probabilities and rates interpreted relative to five-year observation period. MM – Multimorbidity

Additionally, there were significant interactions between multimorbidity and race/ethnicity and socioeconomic status (Fig. 3; Additional File 7). Predicted five-year PHC utilisation rates were higher in individuals with white race/ethnicity, lower educational attainment and those in recipient of welfare. Multimorbidity was associated with higher predicted hospitalisation rates among those with lower educational attainment and welfare recipients. Multimorbidity was associated with a greater risk of death among black Brazilians, those with lower educational attainment and welfare recipients.

**Fig. 4:**
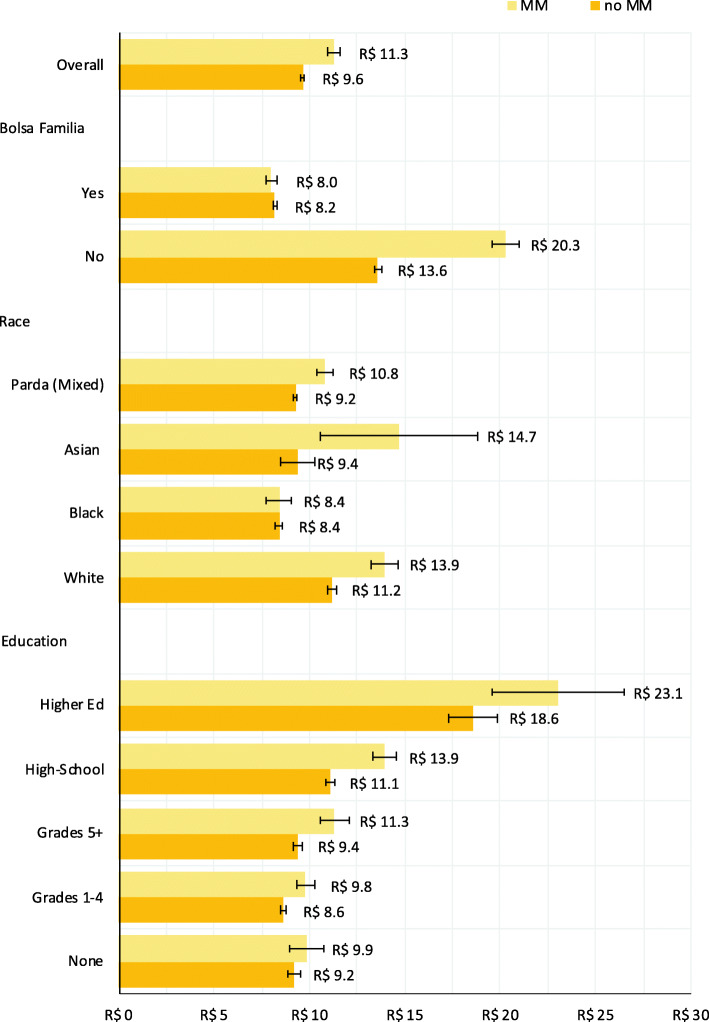
Predicted monthly household expenditures on medicines. Predicted monthly expenditures obtained from adjusted linear regression model including age, sex, race, insurance status, education, Bolsa Família recipient status, multimorbidity diagnosis, and three interactions between multimorbidity status and education, race, and Bolsa Família recipient status

In the subgroup regression analysis of 881,632 individuals with available data, multimorbid individuals spent $R 2.3 (95%CI: 1.9,2.7) more per month than those without multimorbidity (Additional File 8). The association of multimorbidity and higher expenditures on medicines was found across socioeconomic groups (Fig. 4), but the magnitude of increase varied. Individuals in black racial/ethnic, lower educational attainment and welfare recipients had smaller increases in expenditure associated with multimorbidity.

#### Sensitivity analyses

The prevalence of chronic conditions was on average twice as high among PHC users when compared to the whole registered population (Additional File 2). Prevalence estimates for only PHC users (1,722,477) showed very similar patterns across race/ethnicity and socioeconomic groups (Additional File 9). There were also similar effect estimates for risk of hospitalisation and death when examining PHC users only (Additional File 9). Multiple imputation of missing education data did not affect the main results (Additional File 10).

## Discussion

This study analyses multimorbidity and disparities using a large clinical dataset of three million individuals with 13.5 million linked healthcare records – a first for a middle-income country. More than one quarter of registered individuals had a chronic condition and 13% had multimorbidity. Multimorbidity prevalence increased with age: 17% for those aged 18 years and over, 26% for those aged 40 and over, and 37% those age 60 and over. Hypertension and diabetes were leading conditions. Disparities were pervasive with higher rates of multimorbidity and mortality in those with black race/ethnicity, lower educational attainment and those in receipt of welfare.

The high burden of hypertension and diabetes identified in this study is concordant with Brazilian and international studies [33, 34], and the association between multimorbidity and hospitalisations, death and health expenditures is supported by recent systematic reviews [6, 35]. The race/ethnicity group and wider socioeconomic patterning of multimorbidity is also concordant with wider literature [2, 8–14, 24, 28, 34]. In this study, Bolsa Família receipt, lower education and belonging to a black or pardo race/ethnic groups are proxies for lower socioeconomic status. Lower socioeconomic status drives the accumulation of chronic conditions through poorer living standards and diets, lower healthcare access, exposure to pollutants and environmental stressors, diminished levels of health literacy and increased likelihood of unhealthier behaviours such as smoking, alcohol consumption, and low physical activity [36]. In Rio de Janeiro, there is additionally increased neighbourhood violence [37] and national issues related to structural racism and restricted employment and educational opportunities [38]. The results indicated that Black Brazilians had generally higher rates of multimorbidity, lower rates of PHC utilisation, and a higher risk of death than white Brazilians suggesting greater exposure to underlying risk factors and increased barriers to accessing healthcare [39, 40].

In this study, multimorbid individuals with black race/ethnicity and socioeconomically deprived had higher healthcare use and mortality. Little is known in LMICs about which factors increase mortality among individuals with multiple chronic conditions. Studies from the UK suggest behavioural factors are important [41, 42], but in Brazil and other LMICs deep social inequalities and barriers to accessing high-quality (and preventative) healthcare are likely also contributors. The finding that multimorbidity is associated with higher expenditures on medicines is concordant with previous evidence [35]. However, multimorbidity-associated expenditures were greater in higher socioeconomic groups perhaps indicating forgone medicine consumption (due to cost) in lower socioeconomic groups or increased discretionary spend in higher socioeconomic groups where medicines are subsidised [43].

The findings from this study are pertinent to policymakers and clinicians in Brazil and other LMICs. Firstly, multimorbidity is highly prevalent, although likely underreported in clinical records – especially for conditions such as depression and chronic back pain. The estimated prevalence of multimorbidity in this study is lower than others in Brazil (22% of adults nationally [26, 27] and 68% for those over 50 years [28]), and differences may be due to over-reporting in surveys and under-reporting in clinical records. Secondly, disparities in multimorbidity reflect wider patterns of structural disadvantage and deprivation. Addressing inadequate access to high quality healthcare among deprived groups is vital [44, 45], in addition to addressing wider social determinants of multimorbidity [46]. Thirdly, attention to reducing medicine costs in individuals with multimorbidity, especially among those with limited ability to pay, is an important priority. Finally, racial/ethnic inequalities persisted following statistical adjustment for socioeconomic indicators suggesting race/ethnicity may independently affect health outcomes - particularly the worse outcomes for black Brazilians. Given there are almost no biological explanations for these inequalities, this may highlight issues of racial divisions and structural racism.

Using clinical diagnoses from medical records is a major advancement for LMIC studies. Our study has sufficient statistical power to examine disparities in granular detail – not possible in previous studies [45]. However, there are also limitations to consider. PHC-registered individuals may not use PHC (perhaps seeking alternative providers or forgoing healthcare) and therefore the estimated multimorbidity prevalence is likely an underestimate of the true prevalence. Additionally, diagnoses are determined from electronic medical records and the low prevalence of conditions such as chronic back pain and depression may be due to healthcare access issues or poor recording. Deprived populations experience greater barriers to healthcare suggesting underestimates are greater in deprived groups. The study population only covers PHC-registered individuals (just under half the population of Rio), and although the non-PHC covered population is likely to be wealthier due to PHC roll out in poorer areas, extrapolating findings from this study to the whole city is inappropriate. Also, data on race and educational attainment is self-reported with potential for misclassification [47], but biases are unlikely to be associated with multimorbidity or sufficiently large to negate the findings. As educational attainment was determined at the individual-level (as opposed to household or parental level), there may have been biases from including children in the analysis (because their educational attainment was related to their current schooling). However, the prevalence of multimorbidity was very low for children (less than 1.5%) and omitting children from analysis did not alter the results substantially. Moreover, multimorbidity was assessed cross-sectionally without the timing of diagnoses or time until outcomes accounted for. Better understanding of the temporal nature of multimorbidity, associated outcomes and mediating factors is an area for future work – especially in LMICs. This includes using longitudinal data to model multimorbidity over the life course and compare these between countries and settings. Another limitation was the large missing data for education, but sensitivity analyses suggest limited impact on the findings. Lastly, other socioeconomic factors such as income, wealth and housing were not available to analyse and could provide better understanding of the socioeconomic patterning of multimorbidity in Brazil.

## Conclusions

Multimorbidity is prevalent and socioeconomically patterned in Brazil with higher multimorbidity and associated healthcare usage and mortality greater among more deprived socioeconomic groups. Interventions to better prevent and manage multimorbidity and underlying disparities in LMICs are needed. This includes actions targeted towards lower socioeconomic and racial/ethnic groups who have greater exposure to risk factors for chronic conditions and who experience higher barriers to accessing healthcare.

## Supplementary Information

**Additional file 1.** Chronic conditions with ICD-10 and primary care procedure Codes.

**Additional file 2.** Individuals with diagnosed chronic conditions and prevalence estimates.

**Additional file 3.** Chronic conditions ranked by number of diagnosed individuals for multimorbid individuals.

**Additional file 4.** Leading combinations of chronic conditions in multimorbid individuals by contribution to multimorbid mortality and hospitalisations.

**Additional file 5.** Prevalence of multimorbidity by demographic and socioeconomic groups for those aged 45–64 years and 65 year or more.

**Additional file 6.** Logistic regression results on likelihood of any chronic condition or multimorbidity.

**Additional file 7.** Supplementary regression results with socioeconomic interactions.

**Additional file 8.** Linear regression results on household expenditures on medicines (subsample).

**Additional file 9.** Sensitivity analysis showing regression results for PHC users only.

**Additional file 10.** Sensitivity analysis with results from multiple imputation.

## Data Availability

The datasets analysed in this study were generated from linking routine healthcare and administrative datasets. Publicly-available datasets (anonymised and often aggregated) are available from Brazilian government websites: http://tabnet.datasus.gov.br/ and https://www.gov.br/pt-br/servicos/solicitar-cessao-de-dados-identificados-do-cadastro-unico. The specific versions of the datasets used in this analysis (individual-level records with names and tax numbers were linkage) were obtained from the Secretariat for Health in Rio de Janeiro. These linked datasets which were analysed in this study are not publicly available due the confidentiality and sensitivity of the linked individual-level data. However, the corresponding author is available to assist other researchers requesting approval from Brazilian authorities and obtaining appropriate ethical approval for re-use of these datasets.

## Abbreviations

AOR: Adjusted Odds Ratio
ARR: Adjusted Rate Ratio
ICD: International classification of diseases
LMIC: low- and middle-income country
NCD: noncommunicable disease
PHC: primary healthcare

## References

[1] Global Burden of Disease Results Tool [http://ghdx.healthdata.org/gbd-results-tool]. Accessed 17 Apr 2020.

[2] Bloom DE, Cafiero E, Jané-Llopis E, Abrahams-Gessel S, Bloom LR, Fathima S, Feigl AB, Gaziano T, Hamandi A, Mowafi M (2012). The global economic burden of noncommunicable diseases. Program on the global demography of Aging.

[3] Nolte E, McKee M: Caring for people with chronic conditions: a health system perspective: McGraw-Hill International; 2008.

[4] Fortin M, Lapointe L, Hudon C, Vanasse A, Ntetu AL, Maltais D (2004). Multimorbidity and quality of life in primary care: a systematic review. Health Qual Life Outcomes.

[5] Marengoni A, Angleman S, Melis R, Mangialasche F, Karp A, Garmen A, Meinow B, Fratiglioni L (2011). Aging with multimorbidity: a systematic review of the literature. Ageing Res Rev.

[6] Nunes BP, Flores TR, Mielke GI, Thumé E, Facchini LA (2016). Multimorbidity and mortality in older adults: a systematic review and meta-analysis. Arch Gerontol Geriatr.

[7] Pathirana TI, Jackson CA (2018). Socioeconomic status and multimorbidity: a systematic review and meta-analysis. Aust N Z J Public Health.

[8] Rocca WA, Boyd CM, Grossardt BR, Bobo WV, Finney Rutten LJ, Roger VL, Ebbert JO, Therneau TM, Yawn BP, St Sauver JL (2014). Prevalence of multimorbidity in a geographically defined American population: patterns by age, sex, and race/ethnicity. Mayo Clin Proc.

[9] Quiñones AR, Botoseneanu A, Markwardt S, Nagel CL, Newsom JT, Dorr DA, Allore HG (2019). Racial/ethnic differences in multimorbidity development and chronic disease accumulation for middle-aged adults. PLoS One.

[10] Gebregziabher M, Ward RC, Taber DJ, Walker RJ, Ozieh M, Dismuke CE, Axon RN, Egede LE (2018). Ethnic and geographic variations in multimorbidty: evidence from three large cohorts. Soc Sci Med.

[11] Johnson-Lawrence V, Zajacova A, Sneed R (2017). Education, race/ethnicity, and multimorbidity among adults aged 30-64 in the National Health Interview Survey. SSM Popul Health.

[12] Mathur R, Hull SA, Badrick E, Robson J (2011). Cardiovascular multimorbidity: the effect of ethnicity on prevalence and risk factor management. Br J Gen Pract.

[13] Verest WJGM, Galenkamp H, Spek B, Snijder MB, Stronks K, van Valkengoed IGM (2019). Do ethnic inequalities in multimorbidity reflect ethnic differences in socioeconomic status? The HELIUS study. Eur J Pub Health.

[14] Arokiasamy P, Uttamacharya U, Jain K, Biritwum RB, Yawson AE, Wu F, Guo Y, Maximova T, Espinoza BM, Rodríguez AS (2015). The impact of multimorbidity on adult physical and mental health in low- and middle-income countries: what does the study on global ageing and adult health (SAGE) reveal?. BMC Med.

[15] Lee JT, Hamid F, Pati S, Atun R, Millett C (2015). Impact of noncommunicable disease multimorbidity on healthcare utilisation and out-of-pocket expenditures in middle-income countries: cross sectional analysis. PLoS One.

[16] Afshar S, Roderick PJ, Kowal P, Dimitrov BD, Hill AG (2015). Multimorbidity and the inequalities of global ageing: a cross-sectional study of 28 countries using the world health surveys. BMC Public Health.

[17] Barnett K, Mercer SW, Norbury M, Watt G, Wyke S, Guthrie B (2012). Epidemiology of multimorbidity and implications for health care, research, and medical education: a cross-sectional study. Lancet.

[18] Prados-Torres A, Calderón-Larrañaga A, Hancco-Saavedra J, Poblador-Plou B, van den Akker M (2014). Multimorbidity patterns: a systematic review. J Clin Epidemiol.

[19] Academy of Medical Sciences: Multimorbidity: a priority for global health research. In*.*: Academy of Medical Sciences London; 2018.

[20] Castro MC, Massuda A, Almeida G, Menezes-Filho NA, Andrade MV, de Souza Noronha KVM, Rocha R, Macinko J, Hone T, Tasca R, Giovanella L, Malik AM, Werneck H, Fachini LA, Atun R (2019). Brazil's unified health system: the first 30 years and prospects for the future. Lancet.

[21] PNAD Education 2019: More than half of the persons aged 25 and over did not finish high school [https://agenciadenoticias.ibge.gov.br/en/agencia-press-room/2185-news-agency/releases-en/28289-pnad-education-2019-more-than-half-of-the-persons-aged-25-and-over-did-not-finish-high-school].

[22] Instituto Brasileiro de Geografia e Estatística: Desigualdades sociais por cor ou raça no Brasil. Estudos e Pesquisas-Informação Demográfica e Socioeconômica 2019, 41.

[23] Massuda A, Hone T, Leles FAG, de Castro MC, Atun R. The Brazilian health system at crossroads: progress, crisis and resilience. BMJ Global Health. 2018;3(4):e000829. 10.1136/bmjgh-2018-000829. 10.1136/bmjgh-2018-000829 PMC6035510 29997906

[24] Macinko J, Harris MJ (2015). Brazil's family health strategy — delivering community-based primary care in a universal health system. N Engl J Med.

[25] Marinho F, de Azeredo Passos VM, Carvalho Malta D, Barboza França E, Abreu DMX, Araújo VEM, Bustamante-Teixeira MT, Camargos PAM, da Cunha CC, Duncan BB, Felisbino-Mendes MS, Guerra MR, Guimaraes MDC, Lotufo PA, Marcenes W, Oliveira PPV, de Moares Pedroso M, Ribeiro AL, Schmidt MI, Teixeira RA, Vasconcelos AMN, Barreto ML, Bensenor IM, Brant LCC, Claro RM, Costa Pereira A, Cousin E, Curado MP, dos Santos KPB, Faro A, Ferri CP, Furtado JM, Gall J, Glenn SD, Goulart AC, Ishitani LH, Kieling C, Ladeira RM, Machado IE, Martins SCO, Martins-Melo FR, Melo APS, Miller-Petrie MK, Mooney MD, Nunes BP, Palone MRT, Pereira CC, Rasella D, Ray SE, Roever L, de Freitas Saldanha R, Santos IS, Schneider IJC, Santos Silva DA, Silveira DGA, Soares Filho AM, Moraes Sousa TC, Szwarcwald CL, Traebert J, Velasquez-Melendez G, Wang YP, Lozano R, Murray CJL, Naghavi M (2018). Burden of disease in Brazil, 1990–2016: a systematic subnational analysis for the global burden of disease study 2016. Lancet.

[26] Nunes BP, Chiavegatto Filho ADP, Pati S, Cruz Teixeira DS, Flores TR, Camargo-Figuera FA, Munhoz TN, Thumé E, Facchini LA, Rodrigues Batista SR (2017). Contextual and individual inequalities of multimorbidity in Brazilian adults: a cross-sectional national-based study. BMJ Open.

[27] Carvalho JNd, Roncalli ÂG, Cancela MdC, Souza DLBd: Prevalence of multimorbidity in the Brazilian adult population according to socioeconomic and demographic characteristics. PLoS One 2017, 12(4):e0174322, DOI: 10.1371/journal.pone.0174322. 10.1371/journal.pone.0174322 PMC5383049 28384178

[28] Nunes BP, Batista SRR, Andrade FBd, Souza junior PRBd, Lima-Costa MF, Facchini LA: multimorbidity: the Brazilian longitudinal study of aging (ELSI-Brazil). Rev Saude Publica 2018, 52. 10.11606/S1518-8787.2018052000637 PMC6254906 30379288

[29] Hone T, Saraceni V, Medina Coeli C, Trajman A, Rasella D, Millett C, Durovni B: Primary healthcare expansion and mortality in Brazil’s urban poor: A cohort analysis of 1.2 million adults. PLOS Med 2020, 17(10):e1003357. 10.1371/journal.pmed.1003357 PMC7598481 33125387

[30] Coeli CM, Saraceni V, Medeiros PM, et al. Record linkage under suboptimal conditions for data-intensive evaluation of primary care in Rio de Janeiro, Brazil. BMC Med Inform Decis Mak. 2021;21:190. 10.1186/s12911-021-01550-6. 10.1186/s12911-021-01550-6 PMC8204416 34130670

[31] Tonelli M, Wiebe N, Fortin M, Guthrie B, Hemmelgarn BR, James MT, Klarenbach SW, Lewanczuk R, Manns BJ, Ronksley P *et al*: Methods for identifying 30 chronic conditions: application to administrative data. BMC Med Informatics Dec Making 2015, 15(1):31. 10.1186/s12911-015-0155-5 PMC4415341 25886580

[32] Koller D, Schön G, Schäfer I, Glaeske G, van den Bussche H, Hansen H (2014). Multimorbidity and long-term care dependency—a five-year follow-up. BMC Geriatr.

[33] Malta DC, Stopa SR, Szwarcwald CL, Gomes NL, Silva Júnior JB (2015). Reis AACd: surveillance and monitoring of major chronic diseases in Brazil-National Health Survey, 2013. Revista Brasileira de Epidemiologia.

[34] Violan C, Foguet-Boreu Q, Flores-Mateo G, Salisbury C, Blom J, Freitag M, Glynn L, Muth C, Valderas JM (2014). Prevalence, determinants and patterns of multimorbidity in primary care: a systematic review of observational studies. PLoS One.

[35] Sum G, Hone T, Atun R, Millett C, Suhrcke M, Mahal A, Koh GC-H, Lee JT (2018). Multimorbidity and out-of-pocket expenditure on medicines: a systematic review. BMJ Glob Health.

[36] Paixão MJ, Rossetto I, Montovanele F, Carvano LM (2010). Relatório anual das desigualdades raciais no Brasil, 2009–10.

[37] Monteiro J, Rocha R (2017). Drug battles and school achievement: evidence from Rio de Janeiro's favelas. Rev Econ Stat.

[38] Telles EE (2004). Race in another America: the significance of skin color in Brazil: Princeton University press.

[39] Macinko J, Mullachery P, Proietti FA, Lima-Costa MF: Who experiences discrimination in Brazil? Evidence from a large metropolitan region. Int J Equity Health 2012, 11(1):1, 80, DOI: 10.1186/1475-9276-11-80. 10.1186/1475-9276-11-80 PMC3542078 23249451

[40] Constante HM, Bastos JL. Mapping the margins in health services research: how does race intersect with gender and socioeconomic status to shape difficulty accessing HealthCare among unequal Brazilian states? Int J Health Serv. 2020;0020731420979808. 10.1177/0020731420979808 33323017

[41] Dugravot A, Fayosse A, Dumurgier J, Bouillon K, Rayana TB, Schnitzler A, Kivimaki M, Sabia S, Singh-Manoux A (2020). Social inequalities in multimorbidity, frailty, disability, and transitions to mortality: a 24-year follow-up of the Whitehall II cohort study. Lancet Public Health.

[42] Singh-Manoux A, Fayosse A, Sabia S, Tabak A, Shipley M, Dugravot A, Kivimäki M (2018). Clinical, socioeconomic, and behavioural factors at age 50 years and risk of cardiometabolic multimorbidity and mortality: a cohort study. PLoS Med.

[43] Emmerick ICM, Campos MR, da Silva RM, Chaves LA, Bertoldi AD, Ross-Degnan D, Luiza VL (2020). Hypertension and diabetes treatment affordability and government expenditures following changes in patient cost sharing in the “Farmácia popular” program in Brazil: an interrupted time series study. BMC Public Health.

[44] Bastos ML, Menzies D, Hone T, Dehghani K, Trajman A (2017). The impact of the Brazilian family health on selected primary care sensitive conditions: a systematic review. PLoS One.

[45] Hone T, Rasella D, Barreto ML, Majeed A, Millett C (2017). Association between expansion of primary healthcare and racial inequalities in mortality amenable to primary care in Brazil: a national longitudinal analysis. PLoS Med.

[46] Hone T, Macinko J, Millett C (2018). Revisiting Alma-Ata: what is the role of primary health care in achieving the sustainable development goals?. Lancet.

[47] Travassos C, Williams DR. The concept and measurement of race and their relationship to public health: a review focused on Brazil and the United States. Cadernos de Saúde Pública. 2004;20(3):660–78. 10.1590/S0102-311X2004000300003. 10.1590/s0102-311x2004000300003 15263977

