# Supplementary material for: Racial and socioeconomic disparities in multimorbidity and associated healthcare utilisation and outcomes in Brazil: a cross-sectional analysis of three million individuals
Source: BMC Public Health. 2021 Jul 1;21:1287. doi: 10.1186/s12889-021-11328-0 (PMC8252284; doi:10.1186/s12889-021-11328-0)
Supplement: Supplementary file 1 — Additional file 1. Chronic conditions with ICD-10 and primary care procedure Codes. [file 12889_2021_11328_MOESM1_ESM.docx]

**Additional File 1 – Chronic conditions with ICD-10 and primary care procedure Codes**

|  | Condition | ICD10 Codes | ICPC Codes | Criteria |
| --- | --- | --- | --- | --- |
| 1 | **Alcohol misuse** | E52 F10 T51 Z50.2 Z71.4 Z72.1 | T91 P15 P16 | Any code ever recorded |
| 2 | **Allergy** | H01.1 J30 L23 L27.2 L56.4 K52.2 K90.0 T78.1 T78.4 T88.7 | R97 S88 | Any code recorded in last 5 years |
| 3 | **Anaemia** | D50–D53 D55–D58 D59.0–D59.2 D59.4–D59.9 D60.0 D60.8 D60.9 D61 D63–D64 | B78 B80 B81 B82 | Any code recorded in last 5 years |
| 4 | **Anxiety** | F40–F41 | P74 P76 P79 | Any code recorded in last 5 years |
| 5 | **Asthma** | J45-J46 | R96 | Any code ever recorded |
| 6 | **Atherosclerosis/ Peripheral vascular disease** | I65–I66 I67.2 I70 I73.9 | K92 | Any code ever recorded |
| 7 | **Cancer** | C00–C14 C15–C26 C30–C39 C40–C41 C43–C44 C45–C49 C50 C51–C58 C60–C63 C64–C68 C69–C72 C73–C75 C81–C96 C76–C80 C97 D00–D09 D37–D48 | B74 B75 D74 D75 D76 D77 D78 F74 K72 H75 L71 L97 N74 N75 R84 R85 R86 R92 S77 S79 T71 T72 T73 U75 U76 U77 U78 U79 W72 W73 X75 X76 X77 X80 X81 Y77 Y78 Y79 | Any code recorded in last 5 years |
| 8 | **Cardiac arrhythmias** | I44–I45 I46.0 I46.9 I47–I48 I49.1–I49.9 | K78 K79 K80 K84 | Any code ever recorded |
| 9 | **Cardiac valve disorders** | I34–I37 | K83 | Any code ever recorded |
| 10 | **Cerebral ischemia/chronic stroke** | I60–I64 I69 G45 | K89 K90 K91 | Any code ever recorded |
| 11 | **Chronic cholecystitis/gallstones** | K80 K81.1 | D98 | Any code ever recorded |
| 12 | **Chronic gastritis/ Peptic Ulcer disease/ GERD** | K21 K25.4–K25.9 K26.4–K26.9 K27.4–K27.9 K28.4–K28.9 K29.2–K29.9 | D84 D85 D86 D87 | Any code recorded in last 5 years |
| 13 | **Chronic kidney disease** | N00-N23 | U70 U88 U95 U99 | Any code ever recorded |
| 14 | **Chronic low back pain** | M40–M45 M47 M48.0–M48.2 M48.5–M48.9 M50–M54 | L01 L02 L03 L83 L84 L85 L86 L88 L94 L99 | Recorded of any code on separate occasions (not necessarily the same code) in last two years |
| 15 | **COPD** | J40–J44 J47 | R78 R79 R95 | Any code ever recorded |
| 16 | **Dementia** | F00–F03 F05.1 G30 G31 R54 | P70 P05 | Any code ever recorded |
| 17 | **Depression** | F32–F33 F34.1 | P76 | Any code recorded in last two years |
| 18 | **Diabetes mellitus** | E10–E14 | T89 T90 | Any code ever recorded |
| 19 | **Dizziness** | H81–H82 R42 | H82 | Any code recorded in last 2 years |
| 20 | **Epilepsy** | G40-G41 | N88 | Any code ever recorded |
| 21 | **Gynecological problems** | N81 N84–N90 N93 N95 | X85 X86 X87 X99 | Recorded of any code on separate occasions (not necessarily the same code) in last two years |
| 22 | **Heart failure** | I09 I25 I42 I43 I50 | K71 K73 K76 K77 K84 | Any code ever recorded |
| 23 | **Hemorrhoids** | I84 K64 | K96 | Recorded of any code on separate occasions (not necessarily the same code) in last two years |
| 24 | **HIV** | B20-B24 R75 Z71.7 | B90 | Any code ever recorded |
| 25 | **Hypertension** | I10–I15 | K86 K87 | Any code ever recorded |
| 26 | **Hyperuricemia/gout** | E79 M10 | T92 | Any code ever recorded |
| 27 | **Hypotension** | I95 | K88 | Any code ever recorded |
| 28 | **Inflammatory bowel disease** | K50 K51 | D94 | Any code ever recorded |
| 29 | **Insomnia** | G47 F51 | P06 | Any code recorded in last 2 years |
| 30 | **Intestinal diverticulosis** | K57 | D92 | Any code ever recorded |
| 31 | **Joint arthrosis** | M15–M19 | L89 L90 L91 L92 | Any code ever recorded |
| 32 | **Lipid metabolism disorders** | E78 | T93 | Any code ever recorded |
| 33 | **Liver disease** | K70 K71.3–K71.5 K71.7 K72.1 K72.7 K72.9 K73–K74 K76 | D97 | Any code ever recorded |
| 34 | **Lower limb varicosis** | I83 I87.2 | K95 S97 K94 | Any code ever recorded |
| 35 | **Migraine/chronic headache** | G43 G44 | N89 N90 | Any code recorded in last 5 years |
| 36 | **Multiple sclerosis** | G35 G36 G37 H46 | N86 | Any code ever recorded |
| 37 | **Myocardial infarction** | I21-I22 | K75 | Any code ever recorded |
| 38 | **Neuropathies** | G50–G64 | N91 N92 N93 N94 N99 | Any code recorded in last 2 years |
| 39 | **Obesity** | E66 | T82 T83 | Any code recorded in last 5 years |
| 40 | **Osteoporosis** | M80–M82 | L95 | Any code ever recorded |
| 41 | **Parkinson’s disease** | G20–G22 | N87 | Any code ever recorded |
| 42 | **Prostatic hyperplasia** | N40 | Y85 | Any code ever recorded |
| 43 | **Psoriasis** | L40 | S91 | Any code ever recorded |
| 44 | **Rheumatoid arthritis/chronic polyarthritis** | M05–M06 M31.5 M32–M34 M35.1 M35.3 M36.0 M79.0 | L88 | Any code ever recorded |
| 45 | **Schizophrenia** | F20 F21 F23.2 F25 | P72 | Any code ever recorded |
| 46 | **Severe hearing loss** | H90 H91.0 H91.1 H91.3 H91.8 H91.9 | H84 H86 | Any code ever recorded |
| 47 | **Severe vision reduction** | H17–H18 H25–H28 H31 H33 H34.1–H34.2 H34.8–H34.9 H35–H36 H40 H43 H47 H54 | L28 F28 F82 F83 F84 F91 F92 F93 F94 | Any code ever recorded |
| 48 | **Sexual dysfunction** | F52 N48.4 | P07 P08 Y07 | Any code recorded in last 2 years |
| 49 | **Somatoform disorders** | F45 | P75 | Any code ever recorded |
| 50 | **Tuberculosis** | A15-A19 B90 | A70 | Any code recorded in last 2 years |
| 51 | **Thyroid diseases** | E01–E05 E06.1–E06.3 E06.5 E06.9 E07 E89 | T81 T85 T86 | Any code ever recorded |
| 52 | **Tobacco abuse** | F17 | P17 | Any code recorded in last 5 years |
| 53 | **Urinary incontinence** | N39.3–N39.4 R32 | U04 | Any code recorded in last 5 years |

COPD - Chronic obstructive pulmonary disease; GERD - Gastroesophageal Reflux Disease; ICPC - International Classification of Primary Care
