# Supplementary material for: Racial and socioeconomic disparities in multimorbidity and associated healthcare utilisation and outcomes in Brazil: a cross-sectional analysis of three million individuals
Source: BMC Public Health. 2021 Jul 1;21:1287. doi: 10.1186/s12889-021-11328-0 (PMC8252284; doi:10.1186/s12889-021-11328-0)
Supplement: Supplementary file 2 — Additional file 2. Individuals with diagnosed chronic conditions and prevalence estimates. [file 12889_2021_11328_MOESM2_ESM.docx]

**Additional File 2 – Individuals with diagnosed chronic conditions and prevalence estimates**

|  | **All individuals registered with primary care (N (prevalence %))** | **All primary care users (N (prevalence %))** |
| --- | --- | --- |
| Alcohol misuse | 6036 (0.2%) | 5945 (0.3%) |
| Allergy | 74971 (2.5%) | 74950 (4.4%) |
| Anaemia | 30105 (1.0%) | 29564 (1.7%) |
| Anxiety | 38836 (1.3%) | 38836 (2.3%) |
| Asthma | 25872 (0.9%) | 25599 (1.5%) |
| Atherosclerosis/ Peripheral vascular disease | 3650 (0.1%) | 3439 (0.2%) |
| Cancer | 35036 (1.2%) | 30323 (1.8%) |
| Cardiac arrhythmias | 11752 (0.4%) | 11261 (0.7%) |
| Cardiac valve disorders | 1857 (0.1%) | 1796 (0.1%) |
| Cerebral ischemia/chronic stroke | 18317 (0.6%) | 17061 (1.0%) |
| Chronic cholecystitis/gallstones | 23654 (0.8%) | 21287 (1.2%) |
| Chronic gastritis/ Peptic Ulcer disease/GERD | 40699 (1.3%) | 40571 (2.4%) |
| Chronic kidney disease | 29432 (1.0%) | 27521 (1.6%) |
| Chronic low back pain | 21202 (0.7%) | 21201 (1.2%) |
| COPD | 19952 (0.7%) | 19753 (1.1%) |
| Dementia | 9153 (0.3%) | 9064 (0.5%) |
| Depression | 20158 (0.7%) | 20104 (1.2%) |
| Diabetes mellitus | 150276 (5.0%) | 149698 (8.7%) |
| Dizziness | 13333 (0.4%) | 13333 (0.8%) |
| Epilepsy | 12107 (0.4%) | 11792 (0.7%) |
| Gynaecological problems | 20783 (0.7%) | 20748 (1.2%) |
| Heart failure | 16658 (0.6%) | 15508 (0.9%) |
| Haemorrhoids | 6529 (0.2%) | 6524 (0.4%) |
| HIV | 10845 (0.4%) | 10282 (0.6%) |
| Hypertension | 445690 (14.7%) | 444854 (25.8%) |
| Hyperuricemia/gout | 10455 (0.3%) | 10451 (0.6%) |
| Hypotension | 2094 (0.1%) | 2091 (0.1%) |
| Inflammatory bowel disease | 1122 (0.0%) | 1063 (0.1%) |
| Insomnia | 10708 (0.4%) | 10673 (0.6%) |
| Intestinal diverticulosis | 1914 (0.1%) | 1777 (0.1%) |
| Joint arthrosis | 40457 (1.3%) | 39922 (2.3%) |
| Lipid metabolism disorders | 52991 (1.8%) | 52990 (3.1%) |
| Liver disease | 6242 (0.2%) | 5928 (0.3%) |
| Lower limb varicosis | 41139 (1.4%) | 40859 (2.4%) |
| Migraine/chronic headache | 23315 (0.8%) | 23302 (1.4%) |
| Multiple sclerosis | 506 (0.0%) | 471 (0.0%) |
| Myocardial infarction | 7204 (0.2%) | 6370 (0.4%) |
| Neuropathies | 12610 (0.4%) | 12446 (0.7%) |
| Obesity | 49749 (1.6%) | 49587 (2.9%) |
| Osteoporosis | 6619 (0.2%) | 6617 (0.4%) |
| Parkinson’s disease | 2605 (0.1%) | 2592 (0.2%) |
| Prostatic hyperplasia | 11788 (0.4%) | 11393 (0.7%) |
| Psoriasis | 4869 (0.2%) | 4860 (0.3%) |
| Rheumatoid arthritis/chronic polyarthritis | 7748 (0.3%) | 7671 (0.4%) |
| Schizophrenia | 10686 (0.4%) | 10121 (0.6%) |
| Severe hearing loss | 10094 (0.3%) | 10085 (0.6%) |
| Severe vision reduction | 59108 (2.0%) | 58092 (3.4%) |
| Sexual dysfunction | 2513 (0.1%) | 2508 (0.1%) |
| Somatoform disorders | 918 (0.0%) | 918 (0.1%) |
| Tuberculosis | 11162 (0.4%) | 11072 (0.6%) |
| Thyroid diseases | 31618 (1.0%) | 31464 (1.8%) |
| Tobacco abuse | 7108 (0.2%) | 7107 (0.4%) |

COPD - Chronic obstructive pulmonary disease; GERD - Gastroesophageal Reflux Disease.
