# Supplementary material for: Racial and socioeconomic disparities in multimorbidity and associated healthcare utilisation and outcomes in Brazil: a cross-sectional analysis of three million individuals
Source: BMC Public Health. 2021 Jul 1;21:1287. doi: 10.1186/s12889-021-11328-0 (PMC8252284; doi:10.1186/s12889-021-11328-0)
Supplement: Supplementary file 3 — Additional file 3. Chronic conditions ranked by number of diagnosed individuals for multimorbid individuals. [file 12889_2021_11328_MOESM3_ESM.docx]

**Additional File 3 – Chronic conditions ranked by number of diagnosed individuals for multimorbid individuals**

| Rank | Condition | N individuals with condition | Percentage of multimorbid patients |
| --- | --- | --- | --- |
| 1 | Hypertension | 288163 | 73.9% |
| 2 | Diabetes mellitus | 130418 | 33.5% |
| 3 | Severe vision reduction | 47547 | 12.2% |
| 4 | Lipid metabolism disorders | 45026 | 11.6% |
| 5 | Obesity | 37129 | 9.5% |
| 6 | Joint arthrosis | 34031 | 8.7% |
| 7 | Lower limb varicosis | 32613 | 8.4% |
| 8 | Allergy | 30995 | 8.0% |
| 9 | Anxiety | 29609 | 7.6% |
| 10 | Chronic gastritis/ Peptic Ulcer disease/GERD | 26188 | 6.7% |
| 11 | Thyroid diseases | 24913 | 6.4% |
| 12 | Cancer | 22585 | 5.8% |
| 13 | Chronic kidney disease | 20321 | 5.2% |
| 14 | Anaemia | 18175 | 4.7% |
| 15 | Depression | 16837 | 4.3% |
| 16 | Chronic cholecystitis/gallstones | 16067 | 4.1% |
| 17 | Cerebral ischemia/chronic stroke | 15782 | 4.0% |
| 18 | Chronic low back pain | 15731 | 4.0% |
| 19 | Gynaecological problems | 15656 | 4.0% |
| 20 | Heart failure | 14264 | 3.7% |
| 21 | Asthma | 13793 | 3.5% |
| 22 | Migraine/chronic headache | 12878 | 3.3% |
| 23 | COPD | 12765 | 3.3% |
| 24 | Prostatic hyperplasia | 9888 | 2.5% |
| 25 | Hyperuricemia/gout | 9567 | 2.5% |
| 26 | Dizziness | 9539 | 2.4% |
| 27 | Cardiac arrhythmias | 9516 | 2.4% |
| 28 | Neuropathies | 9362 | 2.4% |
| 29 | Insomnia | 8884 | 2.3% |
| 30 | Dementia | 7529 | 1.9% |
| 31 | Urinary incontinence | 7413 | 1.9% |
| 32 | Severe hearing loss | 7187 | 1.8% |
| 33 | Schizophrenia | 6393 | 1.6% |
| 34 | Myocardial infarction | 6108 | 1.6% |
| 35 | Rheumatoid arthritis/chronic polyarthritis | 6057 | 1.6% |
| 36 | Osteoporosis | 5977 | 1.5% |
| 37 | Epilepsy | 5897 | 1.5% |
| 38 | Tobacco abuse | 5527 | 1.4% |
| 39 | Tuberculosis | 5348 | 1.4% |
| 40 | Liver disease | 5108 | 1.3% |
| 41 | HIV | 4986 | 1.3% |
| 42 | Haemorrhoids | 4855 | 1.2% |
| 43 | Alcohol misuse | 4523 | 1.2% |
| 44 | Atherosclerosis/ Peripheral vascular disease | 3260 | 0.8% |
| 45 | Psoriasis | 3252 | 0.8% |
| 46 | Parkinson’s disease | 2134 | 0.5% |
| 47 | Sexual dysfunction | 2018 | 0.5% |
| 48 | Intestinal diverticulosis | 1595 | 0.4% |
| 49 | Cardiac valve disorders | 1441 | 0.4% |
| 50 | Hypotension | 1335 | 0.3% |
| 51 | Inflammatory bowel disease | 742 | 0.2% |
| 52 | Somatoform disorders | 712 | 0.2% |
| 53 | Multiple sclerosis | 291 | 0.1% |
