# Supplementary material for: Racial and socioeconomic disparities in multimorbidity and associated healthcare utilisation and outcomes in Brazil: a cross-sectional analysis of three million individuals
Source: BMC Public Health. 2021 Jul 1;21:1287. doi: 10.1186/s12889-021-11328-0 (PMC8252284; doi:10.1186/s12889-021-11328-0)
Supplement: Supplementary file 4 — Additional file 4. Leading combinations of chronic conditions in multimorbid individuals by contribution to multimorbid mortality and hospitalisations. [file 12889_2021_11328_MOESM4_ESM.docx]

**Additional File 4 – Leading combinations of chronic conditions in multimorbid individuals by contribution to multimorbid mortality and hospitalisations**

Contribution to multimorbid mortality

|  | Conditions | Percentage of deaths of multimorbid individuals | N deaths |
| --- | --- | --- | --- |
| 1 | Hypertension and diabetes mellitus | 10.0% | 1319 |
| 2 | Hypertension and cerebral ischemia/chronic stroke | 3.7% | 486 |
| 3 | Hypertension and cancer | 3.7% | 484 |
| 4 | Hypertension and heart failure | 1.8% | 243 |
| 5 | Hypertension and dementia | 1.7% | 220 |
| 6 | Hypertension, diabetes mellitus and cerebral ischemia/chronic stroke | 1.5% | 204 |
| 7 | Hypertension, diabetes mellitus and cancer | 1.3% | 170 |
| 8 | Hypertension and chronic kidney disease | 1.2% | 156 |
| 9 | Hypertension, diabetes mellitus and chronic kidney disease | 1.1% | 144 |
| 10 | Hypertension and severe vision reduction | 1.0% | 129 |

Contribution to multimorbid hospitalisations

|  | Conditions | Percentage of hospitalisations of multimorbid individuals | N hospitalisations |
| --- | --- | --- | --- |
| 1 | Hypertension and diabetes mellitus | 4.4% | 6414 |
| 2 | Hypertension and cancer | 2.9% | 4281 |
| 3 | Hypertension and cerebral ischemia/chronic stroke | 1.5% | 2213 |
| 4 | Hypertension and severe vision reduction | 1.3% | 1959 |
| 5 | Hypertension and chronic kidney disease | 1.3% | 1936 |
| 6 | Hypertension and chronic cholecystitis/gallstones | 1.2% | 1764 |
| 7 | Hypertension, diabetes mellitus and cancer | 1.0% | 1497 |
| 8 | Hypertension and heart failure | 0.9% | 1311 |
| 9 | Hypertension, diabetes mellitus and severe vision reduction | 0.9% | 1285 |
| 10 | Hypertension and joint arthrosis | 0.8% | 1121 |
