# Supplementary material for: Racial and socioeconomic disparities in multimorbidity and associated healthcare utilisation and outcomes in Brazil: a cross-sectional analysis of three million individuals
Source: BMC Public Health. 2021 Jul 1;21:1287. doi: 10.1186/s12889-021-11328-0 (PMC8252284; doi:10.1186/s12889-021-11328-0)

**Additional File 5 - Prevalence of multimorbidity by demographic and socioeconomic groups for those aged 45-64 years and 65 year or more**
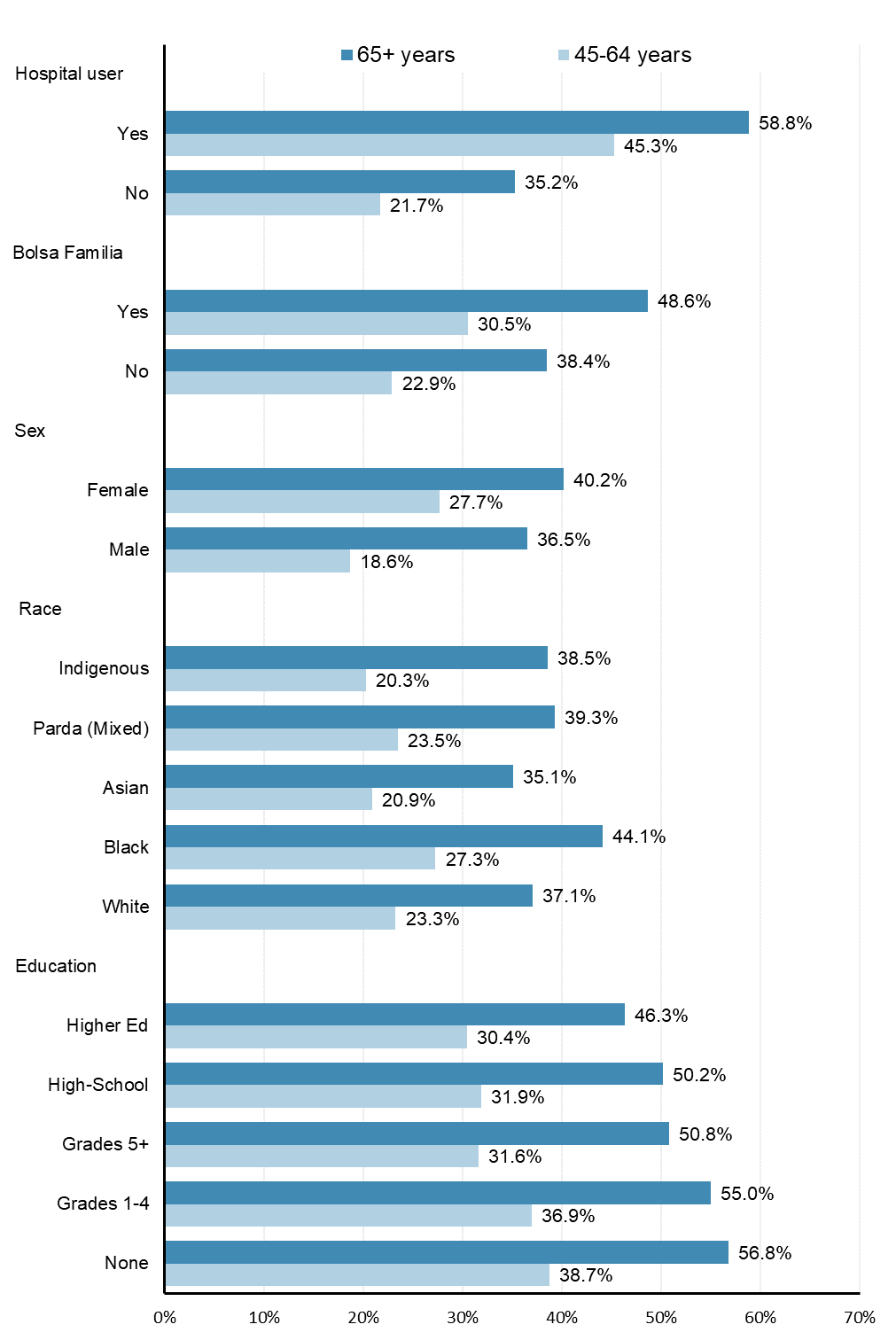

Supplement: Supplementary file 5 — Additional file 5. Prevalence of multimorbidity by demographic and socioeconomic groups for those aged 45–64 years and 65 year or more. [file 12889_2021_11328_MOESM5_ESM.docx]
