# Supplementary material for: Racial and socioeconomic disparities in multimorbidity and associated healthcare utilisation and outcomes in Brazil: a cross-sectional analysis of three million individuals
Source: BMC Public Health. 2021 Jul 1;21:1287. doi: 10.1186/s12889-021-11328-0 (PMC8252284; doi:10.1186/s12889-021-11328-0)
Supplement: Supplementary file 6 — Additional file 6. Logistic regression results on likelihood of any chronic condition or multimorbidity. [file 12889_2021_11328_MOESM6_ESM.docx]

**Additional File 6 – Logistic regression results on likelihood of any chronic condition or multimorbidity**

|  | **Any chronic conditions** | | **Multimorbidity** | |  |
| --- | --- | --- | --- | --- | --- |
|  | **AOR** | **95% CI** | **AOR** | **95% CI** | |
| Sex |  |  |  |  | |
| Male | 1 (ref) | - | 1 (ref) | - | |
| Female | 1.231*** | 1.222,1.240 | 1.394*** | 1.382,1.407 | |
| Race |  |  |  |  | |
| White | 1 (ref) | - | 1 (ref) | - | |
| Black | 1.087*** | 1.074,1.099 | 1.045*** | 1.032,1.059 | |
| Asian | 0.947* | 0.903,0.993 | 0.894*** | 0.845,0.946 | |
| Pardo (Mixed) | 0.968*** | 0.960,0.975 | 0.941*** | 0.933,0.950 | |
| Indigenous | 0.877* | 0.778,0.990 | 0.815** | 0.715,0.929 | |
| Age group |  |  |  |  | |
| 0-4 years | 1 (ref) | - | 1 (ref) | - | |
| 0-9 years | 1.372*** | 1.341,1.404 | 1.625*** | 1.533,1.722 | |
| 10-14 years | 1.640*** | 1.598,1.683 | 2.016*** | 1.900,2.138 | |
| 15-19 years | 1.791*** | 1.746,1.838 | 2.455*** | 2.320,2.597 | |
| 20-24 years | 2.260*** | 2.203,2.318 | 3.611*** | 3.423,3.810 | |
| 25-29 years | 3.004*** | 2.929,3.081 | 5.476*** | 5.198,5.768 | |
| 30-34 years | 4.114*** | 4.013,4.217 | 8.701*** | 8.275,9.148 | |
| 35-39 years | 5.884*** | 5.742,6.029 | 13.204*** | 12.573,13.866 | |
| 40-44 years | 8.702*** | 8.492,8.916 | 20.153*** | 19.202,21.152 | |
| 45-49 years | 12.379*** | 12.081,12.684 | 29.684*** | 28.297,31.139 | |
| 50-54 years | 18.124*** | 17.689,18.569 | 43.308*** | 41.301,45.412 | |
| 55-59 years | 25.511*** | 24.890,26.148 | 58.215*** | 55.525,61.036 | |
| 60-64 years | 34.584*** | 33.720,35.470 | 76.774*** | 73.226,80.493 | |
| 65-69 years | 46.294*** | 45.065,47.555 | 94.993*** | 90.574,99.629 | |
| 70+ years | 57.012*** | 55.649,58.408 | 109.161*** | 104.204,114.353 | |
| Bolsa Família recipient |  |  |  |  | |
| No | 1 (ref) | - | 1 (ref) | - | |
| Yes | 1.068*** | 1.059,1.078 | 1.140*** | 1.126,1.153 | |
| Private health insurance |  |  |  |  | |
| No | 1 (ref) | - | 1 (ref) | - | |
| Yes | 0.796*** | 0.785,0.809 | 0.758*** | 0.744,0.771 | |
| Highest education |  |  |  |  | |
| None/Pre-school/Literacy class | 1 (ref) | - | 1 (ref) | - | |
| Elementary School (Grades 1-4) | 0.961*** | 0.946,0.975 | 0.994 | 0.978,1.009 | |
| Elementary School (Grades 5+) | 0.852*** | 0.838,0.866 | 0.866*** | 0.852,0.882 | |
| High-School | 0.872*** | 0.859,0.886 | 0.868*** | 0.854,0.882 | |
| Higher Education | 0.810*** | 0.792,0.828 | 0.793*** | 0.774,0.811 | |
| Missing | 0.587*** | 0.575,0.598 | 0.521*** | 0.510,0.533 | |
| PHC user |  |  |  |  | |
| No | 1 (ref) | - | 1 (ref) | - | |
| Yes | 86.701*** | 85.306,88.118 | 172.897*** | 164.783,181.411 | |
| Hospital admission |  |  |  |  | |
| No | 1 (ref) | - | 1 (ref) | - | |
| Yes | 4.582*** | 4.510,4.656 | 2.671*** | 2.637,2.706 | |
|  |  |  |  |  | |
| N (individuals) | 3027335 |  | 3027335 |  | |

Results from logistic regression models; PHC - Primary healthcare; AOR – Adjusted Odds Ratio; *p<0.05; **p<0.01; *** p<0.001.
