# Supplementary material for: Racial and socioeconomic disparities in multimorbidity and associated healthcare utilisation and outcomes in Brazil: a cross-sectional analysis of three million individuals
Source: BMC Public Health. 2021 Jul 1;21:1287. doi: 10.1186/s12889-021-11328-0 (PMC8252284; doi:10.1186/s12889-021-11328-0)
Supplement: Supplementary file 7 — Additional file 7. Supplementary regression results with socioeconomic interactions. [file 12889_2021_11328_MOESM7_ESM.docx]

**Additional File 7 – Supplementary regression results with socioeconomic interactions**

**Poisson regression model results on number of PHC consultations**

|  | ARR | 95% CI | ARR | 95% CI |
| --- | --- | --- | --- | --- |
| Sex |  |  |  |  |
| Male | 1 (ref) | - | 1 (ref) | - |
| Female | 1.265*** | 1.260,1.270 | 1.260*** | 1.255,1.264 |
| Race |  |  |  |  |
| White | 1 (ref) | - | 1 (ref) | - |
| Black | 0.989*** | 0.984,0.995 | 1.023*** | 1.016,1.029 |
| Asian (*Amarelo*) | 0.894*** | 0.873,0.914 | 0.897*** | 0.874,0.921 |
| Pardo (Mixed) | 0.978*** | 0.974,0.982 | 0.991*** | 0.987,0.996 |
| Indigenous | 0.920** | 0.869,0.974 | 0.886*** | 0.832,0.944 |
| Age group |  |  |  |  |
| 0-4 years | 1 (ref) | - | 1 (ref) | - |
| 0-9 years | 0.600*** | 0.595,0.605 | 0.605*** | 0.600,0.610 |
| 10-14 years | 0.509*** | 0.504,0.515 | 0.536*** | 0.530,0.542 |
| 15-19 years | 0.620*** | 0.614,0.627 | 0.653*** | 0.646,0.660 |
| 20-24 years | 0.849*** | 0.840,0.858 | 0.888*** | 0.878,0.898 |
| 25-29 years | 0.917*** | 0.907,0.928 | 0.967*** | 0.956,0.978 |
| 30-34 years | 0.903*** | 0.893,0.914 | 0.956*** | 0.945,0.967 |
| 35-39 years | 0.884*** | 0.874,0.895 | 0.934*** | 0.923,0.945 |
| 40-44 years | 0.881*** | 0.871,0.892 | 0.928*** | 0.918,0.939 |
| 45-49 years | 0.914*** | 0.904,0.925 | 0.961*** | 0.950,0.972 |
| 50-54 years | 0.969*** | 0.958,0.980 | 1.017** | 1.005,1.028 |
| 55-59 years | 0.997 | 0.987,1.009 | 1.047*** | 1.036,1.059 |
| 60-64 years | 1.011 | 1.000,1.022 | 1.060*** | 1.048,1.072 |
| 65-69 years | 1.010 | 0.998,1.022 | 1.058*** | 1.046,1.071 |
| 70+ years | 0.952*** | 0.942,0.961 | 0.998 | 0.988,1.008 |
| Bolsa Familia recipient |  |  |  |  |
| No | 1 (ref) | - | 1 (ref) | - |
| Yes | 1.080*** | 1.075,1.084 | 1.026*** | 1.021,1.030 |
| Private health insurance |  |  |  |  |
| No | 1 (ref) | - | 1 (ref) | - |
| Yes | 0.666*** | 0.661,0.671 | 0.681*** | 0.676,0.687 |
| Highest education |  |  |  |  |
| None/Pre-school/Literacy class | 1 (ref) | - | 1 (ref) | - |
| Elementary School (Grades 1-4) | 0.984*** | 0.976,0.991 | 1.023*** | 1.015,1.032 |
| Elementary School (Grades 5+) | 0.735*** | 0.729,0.742 | 0.631*** | 0.625,0.637 |
| High-School | 0.889*** | 0.882,0.896 | 0.885*** | 0.877,0.893 |
| Higher Education | 0.847*** | 0.837,0.857 | 0.828*** | 0.818,0.838 |
| Missing | 0.139*** | 0.138,0.141 | 0.087*** | 0.086,0.088 |
| Multimorbidity |  |  |  |  |
| No | 1 (ref) | - | 1 (ref) | - |
| Yes | 3.457*** | 3.441,3.473 | 3.247*** | 3.203,3.291 |
|  |  |  |  |  |
| Interactions |  |  |  |  |
| Race x multimorbidity |  |  |  |  |
| White | - |  | 1 (ref) | - |
| Black | - |  | 0.926*** | 0.915,0.936 |
| Asian (*Amarelo*) | - |  | 0.993 | 0.945,1.042 |
| Pardo (Mixed) | - |  | 0.965*** | 0.956,0.973 |
| Indigenous | - |  | 1.096 | 0.977,1.230 |
| Education x multimorbidity |  |  |  |  |
| None/Pre-school/Literacy class | - |  | 1 (ref) | - |
| Elementary School (Grades 1-4) | - |  | 0.911*** | 0.899,0.924 |
| Elementary School (Grades 5+) | - |  | 1.344*** | 1.323,1.364 |
| High-School | - |  | 0.970*** | 0.956,0.984 |
| Higher Education | - |  | 1.004 | 0.982,1.027 |
| Missing | - |  | 7.056*** | 6.916,7.198 |
| Bolsa Familia recipient x multimorbidity |  |  |  |  |
| No | - |  | 1 (ref) | - |
| Yes | - |  | 1.129*** | 1.118,1.141 |
|  |  |  |  |  |
| N | 3027335 |  | 3027335 |  |

PHC - Primary healthcare; ARR – Adjusted Rate Ratios. Robust standard errors used. *p<0.05; **p<0.01; *** p<0.001.

**Poisson regression model results on number of hospitalisations**

|  | ARR | 95% CI | ARR | 95% CI |
| --- | --- | --- | --- | --- |
| Sex |  |  |  |  |
| Male | 1 (ref) | - | 1 (ref) | - |
| Female | 0.703*** | 0.693,0.713 | 0.702*** | 0.692,0.712 |
| Race |  |  |  |  |
| White | 1 (ref) | - | 1 (ref) | - |
| Black | 1.010 | 0.988,1.033 | 1.034* | 1.005,1.063 |
| Asian (*Amarelo*) | 1.182** | 1.043,1.340 | 1.189* | 1.022,1.384 |
| Pardo (Mixed) | 0.989 | 0.974,1.004 | 0.984 | 0.965,1.004 |
| Indigenous | 0.916 | 0.784,1.070 | 0.892 | 0.741,1.074 |
| Age group |  |  |  |  |
| 0-4 years | 1 (ref) | - | 1 (ref) | - |
| 0-9 years | 1.067*** | 1.033,1.102 | 1.089*** | 1.054,1.125 |
| 10-14 years | 0.730*** | 0.697,0.765 | 0.799*** | 0.753,0.847 |
| 15-19 years | 0.623*** | 0.593,0.655 | 0.699*** | 0.656,0.745 |
| 20-24 years | 0.628*** | 0.597,0.661 | 0.728*** | 0.681,0.777 |
| 25-29 years | 0.769*** | 0.728,0.811 | 0.899** | 0.840,0.963 |
| 30-34 years | 0.917** | 0.866,0.970 | 1.075* | 1.000,1.155 |
| 35-39 years | 1.026 | 0.972,1.083 | 1.196*** | 1.118,1.280 |
| 40-44 years | 1.121*** | 1.063,1.183 | 1.300*** | 1.215,1.391 |
| 45-49 years | 1.249*** | 1.184,1.317 | 1.443*** | 1.351,1.541 |
| 50-54 years | 1.427*** | 1.350,1.507 | 1.642*** | 1.534,1.757 |
| 55-59 years | 1.506*** | 1.432,1.585 | 1.731*** | 1.623,1.845 |
| 60-64 years | 1.649*** | 1.570,1.731 | 1.890*** | 1.775,2.012 |
| 65-69 years | 1.845*** | 1.761,1.933 | 2.113*** | 1.988,2.245 |
| 70+ years | 2.222*** | 2.126,2.322 | 2.551*** | 2.402,2.710 |
| Bolsa Familia recipient |  |  |  |  |
| No | 1 (ref) | - | 1 (ref) | - |
| Yes | 1.341*** | 1.318,1.364 | 1.314*** | 1.288,1.341 |
| Private health insurance |  |  |  |  |
| No | 1 (ref) | - | 1 (ref) | - |
| Yes | 0.536*** | 0.522,0.552 | 0.548*** | 0.533,0.564 |
| Highest education |  |  |  |  |
| None/Pre-school/Literacy class | 1 (ref) | - | 1 (ref) | - |
| Elementary School (Grades 1-4) | 0.844*** | 0.817,0.872 | 0.797*** | 0.756,0.841 |
| Elementary School (Grades 5+) | 0.832*** | 0.803,0.862 | 0.729*** | 0.690,0.771 |
| High-School | 0.809*** | 0.780,0.840 | 0.698*** | 0.658,0.740 |
| Higher Education | 0.754*** | 0.716,0.794 | 0.625*** | 0.581,0.672 |
| Missing | 0.701*** | 0.671,0.732 | 0.546*** | 0.514,0.580 |
| Multimorbidity |  |  |  |  |
| No | 1 (ref) | - | 1 (ref) | - |
| Yes | 2.749*** | 2.689,2.810 | 2.154*** | 2.013,2.305 |
|  |  |  |  |  |
| Interactions |  |  |  |  |
| Race x multimorbidity |  |  |  |  |
| White | - |  | 1 (ref) | - |
| Black | - |  | 0.938** | 0.897,0.981 |
| Asian (*Amarelo*) | - |  | 0.982 | 0.751,1.286 |
| Pardo (Mixed) | - |  | 1.006 | 0.975,1.037 |
| Indigenous | - |  | 1.093 | 0.788,1.516 |
| Education x multimorbidity |  |  |  |  |
| None/Pre-school/Literacy class | - |  | 1 (ref) | - |
| Elementary School (Grades 1-4) | - |  | 1.092** | 1.023,1.165 |
| Elementary School (Grades 5+) | - |  | 1.261*** | 1.176,1.352 |
| High-School | - |  | 1.274*** | 1.187,1.367 |
| Higher Education | - |  | 1.367*** | 1.235,1.512 |
| Missing | - |  | 2.660*** | 2.445,2.894 |
| Bolsa Familia recipient x multimorbidity |  |  |  |  |
| No | - |  | 1 (ref) | - |
| Yes | - |  | 1.019 | 0.982,1.059 |
|  |  |  |  |  |
| N | 3027335 |  | 3027335 |  |

PHC - Primary healthcare; ARR – Adjusted Rate Ratios. Robust standard errors used. *p<0.05; **p<0.01; *** p<0.001.

**Logistic regression model results on likelihood of death**

|  | ARR | 95% CI | ARR | 95% CI |
| --- | --- | --- | --- | --- |
| Sex |  |  |  |  |
| Male | 1 (ref) | - | 1 (ref) | - |
| Female | 0.581*** | 0.569,0.593 | 0.583*** | 0.571,0.595 |
| Race |  |  |  |  |
| White | 1 (ref) | - | 1 (ref) | - |
| Black | 1.166*** | 1.130,1.203 | 1.215*** | 1.169,1.263 |
| Asian (*Amarelo*) | 1.231** | 1.087,1.395 | 1.259** | 1.089,1.455 |
| Pardo (Mixed) | 0.971* | 0.950,0.993 | 0.947*** | 0.922,0.974 |
| Indigenous | 1.106 | 0.832,1.469 | 1.15 | 0.819,1.615 |
| Age group |  |  |  |  |
| 0-4 years | 1 (ref) | - | 1 (ref) | - |
| 0-9 years | 0.243*** | 0.192,0.307 | 0.242*** | 0.191,0.306 |
| 10-14 years | 0.266*** | 0.209,0.338 | 0.270*** | 0.212,0.344 |
| 15-19 years | 1.074 | 0.923,1.250 | 1.105 | 0.945,1.292 |
| 20-24 years | 1.753*** | 1.529,2.011 | 1.814*** | 1.575,2.089 |
| 25-29 years | 1.749*** | 1.523,2.008 | 1.801*** | 1.563,2.076 |
| 30-34 years | 1.820*** | 1.587,2.087 | 1.868*** | 1.623,2.151 |
| 35-39 years | 2.306*** | 2.021,2.630 | 2.360*** | 2.060,2.704 |
| 40-44 years | 3.123*** | 2.746,3.551 | 3.191*** | 2.794,3.644 |
| 45-49 years | 4.069*** | 3.589,4.614 | 4.154*** | 3.648,4.731 |
| 50-54 years | 6.347*** | 5.624,7.162 | 6.479*** | 5.716,7.342 |
| 55-59 years | 9.974*** | 8.862,11.225 | 10.173*** | 9.001,11.497 |
| 60-64 years | 14.854*** | 13.216,16.696 | 15.154*** | 13.427,17.104 |
| 65-69 years | 21.662*** | 19.282,24.335 | 22.105*** | 19.596,24.935 |
| 70+ years | 64.473*** | 57.591,72.178 | 65.831*** | 58.554,74.012 |
| Bolsa Familia recipient |  |  |  |  |
| No | 1 (ref) | - | 1 (ref) | - |
| Yes | 1.517*** | 1.460,1.577 | 1.592*** | 1.517,1.670 |
| Private health insurance |  |  |  |  |
| No | 1 (ref) | - | 1 (ref) | - |
| Yes | 0.820*** | 0.793,0.849 | 0.810*** | 0.783,0.838 |
| Highest education |  |  |  |  |
| None/Pre-school/Literacy class | 1 (ref) | - | 1 (ref) | - |
| Elementary School (Grades 1-4) | 0.788*** | 0.759,0.819 | 0.747*** | 0.706,0.790 |
| Elementary School (Grades 5+) | 0.731*** | 0.700,0.763 | 0.709*** | 0.666,0.754 |
| High-School | 0.593*** | 0.568,0.620 | 0.514*** | 0.483,0.548 |
| Higher Education | 0.546*** | 0.505,0.590 | 0.461*** | 0.412,0.516 |
| Missing | 1.288*** | 1.240,1.338 | 1.302*** | 1.237,1.370 |
| Multimorbidity |  |  |  |  |
| No | 1 (ref) | - | 1 (ref) | - |
| Yes | 1.327*** | 1.293,1.361 | 1.258*** | 1.175,1.348 |
|  |  |  |  |  |
| Interactions |  |  |  |  |
| Race x multimorbidity |  |  |  |  |
| White | - |  | 1 (ref) | - |
| Black | - |  | 0.907** | 0.849,0.969 |
| Asian (*Amarelo*) | - |  | 0.921 | 0.692,1.224 |
| Pardo (Mixed) | - |  | 1.089*** | 1.038,1.142 |
| Indigenous | - |  | 0.877 | 0.473,1.625 |
| Education x multimorbidity |  |  |  |  |
| None/Pre-school/Literacy class | - |  | 1 (ref) | - |
| Elementary School (Grades 1-4) | - |  | 1.100* | 1.020,1.187 |
| Elementary School (Grades 5+) | - |  | 1.052 | 0.965,1.146 |
| High-School | - |  | 1.342*** | 1.231,1.463 |
| Higher Education | - |  | 1.422*** | 1.218,1.660 |
| Missing | - |  | 0.671*** | 0.609,0.739 |
| Bolsa Familia recipient x multimorbidity |  |  |  |  |
| No | - |  | 1 (ref) | - |
| Yes | - |  | 0.903* | 0.834,0.978 |
|  |  |  |  |  |
| N | 3027335 |  | 3027335 |  |

PHC - Primary healthcare; AOR – Adjusted Odds Ratio. Robust standard errors used. *p<0.05; **p<0.01; *** p<0.001.
