# Supplementary material for: Racial and socioeconomic disparities in multimorbidity and associated healthcare utilisation and outcomes in Brazil: a cross-sectional analysis of three million individuals
Source: BMC Public Health. 2021 Jul 1;21:1287. doi: 10.1186/s12889-021-11328-0 (PMC8252284; doi:10.1186/s12889-021-11328-0)
Supplement: Supplementary file 8 — Additional file 8. Linear regression results on household expenditures on medicines (subsample). [file 12889_2021_11328_MOESM8_ESM.docx]

**Additional File 8 – Linear regression results on household expenditures on medicines (subsample)**

|  | ß | 95% CI | ß | 95% CI |
| --- | --- | --- | --- | --- |
| Sex |  |  |  |  |
| Male | 1 (ref) | - | 1 (ref) | - |
| Female | -0.535*** | -0.738,-0.333 | -0.517*** | -0.720,-0.315 |
| Race |  |  |  |  |
| White | 1 (ref) | - | 1 (ref) | - |
| Black | -3.146*** | -3.452,-2.839 | -2.765*** | -3.087,-2.444 |
| Asian (*Amarelo*) | -1.584*** | -2.513,-0.655 | -1.806*** | -2.725,-0.888 |
| Pardo (Mixed) | -2.092*** | -2.361,-1.823 | -1.931*** | -2.216,-1.647 |
| Indigenous | -3.794*** | -5.910,-1.677 | -3.760*** | -5.781,-1.739 |
| Age group |  |  |  |  |
| 0-4 years | 1 (ref) | - | 1 (ref) | - |
| 0-9 years | 0.664*** | 0.326,1.002 | 0.657*** | 0.318,0.995 |
| 10-14 years | 1.252*** | 0.814,1.691 | 1.322*** | 0.882,1.761 |
| 15-19 years | 1.473*** | 0.960,1.986 | 1.619*** | 1.105,2.133 |
| 20-24 years | 0.621* | 0.037,1.204 | 0.886** | 0.302,1.470 |
| 25-29 years | 0.741* | 0.160,1.322 | 1.055*** | 0.471,1.639 |
| 30-34 years | 0.576* | 0.044,1.109 | 0.896** | 0.362,1.431 |
| 35-39 years | 0.825** | 0.273,1.377 | 1.147*** | 0.594,1.701 |
| 40-44 years | 1.559*** | 0.811,2.306 | 1.906*** | 1.153,2.659 |
| 45-49 years | 1.884*** | 1.339,2.429 | 2.230*** | 1.684,2.775 |
| 50-54 years | 3.703*** | 3.100,4.306 | 3.999*** | 3.394,4.604 |
| 55-59 years | 6.781*** | 6.107,7.455 | 6.966*** | 6.289,7.643 |
| 60-64 years | 12.702*** | 11.843,13.561 | 12.756*** | 11.893,13.619 |
| 65-69 years | 17.266*** | 16.079,18.454 | 17.116*** | 15.923,18.309 |
| 70+ years | 32.908*** | 31.669,34.146 | 32.348*** | 31.085,33.610 |
| Bolsa Familia recipient |  |  |  |  |
| No | 1 (ref) | - | 1 (ref) | - |
| Yes | -6.317*** | -6.543,-6.091 | -5.392*** | -5.630,-5.154 |
| Private health insurance |  |  |  |  |
| No | 1 (ref) | - | 1 (ref) | - |
| Yes | 6.922*** | 5.951,7.893 | 6.996*** | 6.024,7.967 |
| Highest education |  |  |  |  |
| None/Pre-school/Literacy class | 1 (ref) | - | 1 (ref) | - |
| Elementary School (Grades 1-4) | -0.396* | -0.754,-0.038 | -0.524** | -0.887,-0.162 |
| Elementary School (Grades 5+) | 0.555** | 0.152,0.957 | 0.253 | -0.158,0.665 |
| High-School | 2.339*** | 1.896,2.782 | 1.915*** | 1.457,2.373 |
| Higher Education | 10.161*** | 8.900,11.421 | 9.453*** | 8.128,10.777 |
| Missing | -3.263*** | -3.951,-2.575 | -3.130*** | -3.829,-2.432 |
| Multimorbidity |  |  |  |  |
| No | 1 (ref) | - | 1 (ref) | - |
| Yes | 2.316*** | 1.944,2.688 | 6.892*** | 5.533,8.251 |
|  |  |  |  |  |
| Interactions |  |  |  |  |
| Race x multimorbidity |  |  |  |  |
| White | - |  | 1 (ref) | - |
| Black | - |  | -2.790*** | -3.800,-1.781 |
| Asian (*Amarelo*) | - |  | 2.556 | -1.695,6.807 |
| Pardo (Mixed) | - |  | -1.251** | -2.128,-0.374 |
| Indigenous | - |  | -0.422 | -9.928,9.085 |
| Education x multimorbidity |  |  |  |  |
| None/Pre-school/Literacy class | - |  | 1 (ref) | - |
| Elementary School (Grades 1-4) | - |  | 0.46 | -0.631,1.550 |
| Elementary School (Grades 5+) | - |  | 1.197 | -0.050,2.445 |
| High-School | - |  | 2.141*** | 0.936,3.345 |
| Higher Education | - |  | 3.739 | -0.090,7.568 |
| Missing | - |  | -6.722*** | -10.088,-3.355 |
| Bolsa Familia recipient x multimorbidity |  |  |  |  |
| No | - |  | 1 (ref) | - |
| Yes | - |  | -6.906*** | -7.720,-6.092 |
|  |  |  |  |  |
| N | 881632 |  | 881632 |  |

PHC - Primary healthcare. Robust standard errors used. *p<0.05; **p<0.01; *** p<0.001.
