# Supplementary material for: Racial and socioeconomic disparities in multimorbidity and associated healthcare utilisation and outcomes in Brazil: a cross-sectional analysis of three million individuals
Source: BMC Public Health. 2021 Jul 1;21:1287. doi: 10.1186/s12889-021-11328-0 (PMC8252284; doi:10.1186/s12889-021-11328-0)
Supplement: Supplementary file 9 — Additional file 9. Sensitivity analysis showing regression results for PHC users only. [file 12889_2021_11328_MOESM9_ESM.docx]

**Additional File 9 - Sensitivity analysis showing regression results for PHC users only**

**Logistic regression on likelihood of multimorbidity and death for PHC users only**

|  | **Multimorbidity** | | **Death** | |  |
| --- | --- | --- | --- | --- | --- |
|  | **AOR** | **95% CI** | **AOR** | **95% CI** | |
| Sex |  |  |  |  | |
| Male | 1 (ref) | - | 1 (ref) | - | |
| Female | 1.398*** | 1.386,1.411 | 0.536*** | 0.521,0.551 | |
| Race |  |  |  |  | |
| White | 1 (ref) | - | 1 (ref) | - | |
| Black | 1.043*** | 1.030,1.057 | 1.077*** | 1.033,1.122 | |
| Asian | 0.896*** | 0.846,0.948 | 1.156 | 0.967,1.383 | |
| Pardo (Mixed) | 0.941*** | 0.932,0.950 | 0.963* | 0.935,0.993 | |
| Indigenous | 0.809** | 0.710,0.922 | 1.061 | 0.725,1.553 | |
| Age group |  |  |  |  | |
| 0-4 years | 1 (ref) | - | 1 (ref) | - | |
| 0-9 years | 1.627*** | 1.535,1.724 | 0.338*** | 0.240,0.475 | |
| 10-14 years | 2.017*** | 1.902,2.140 | 0.420*** | 0.295,0.597 | |
| 15-19 years | 2.462*** | 2.327,2.604 | 1.718*** | 1.374,2.149 | |
| 20-24 years | 3.609*** | 3.420,3.808 | 3.024*** | 2.464,3.711 | |
| 25-29 years | 5.489*** | 5.210,5.782 | 3.566*** | 2.910,4.371 | |
| 30-34 years | 8.708*** | 8.282,9.157 | 4.117*** | 3.379,5.017 | |
| 35-39 years | 13.252*** | 12.618,13.918 | 5.173*** | 4.286,6.244 | |
| 40-44 years | 20.208*** | 19.253,21.211 | 6.912*** | 5.767,8.285 | |
| 45-49 years | 29.793*** | 28.399,31.256 | 8.576*** | 7.189,10.230 | |
| 50-54 years | 43.408*** | 41.394,45.520 | 12.600*** | 10.632,14.933 | |
| 55-59 years | 58.339*** | 55.639,61.170 | 18.423*** | 15.599,21.760 | |
| 60-64 years | 76.844*** | 73.288,80.574 | 25.643*** | 21.746,30.239 | |
| 65-69 years | 94.980*** | 90.554,99.623 | 35.241*** | 29.902,41.533 | |
| 70+ years | 108.906*** | 103.952,114.095 | 94.110*** | 80.166,110.480 | |
| Bolsa Família recipient |  |  |  |  | |
| No | 1 (ref) | - | 1 (ref) | - | |
| Yes | 1.139*** | 1.126,1.153 | 1.103*** | 1.051,1.157 | |
| Private health insurance |  |  |  |  | |
| No | 1 (ref) | - | 1 (ref) | - | |
| Yes | 0.762*** | 0.748,0.776 | 1.028 | 0.975,1.083 | |
| Highest education |  |  |  |  | |
| None/Pre-school/Literacy class | 1 (ref) | - | 1 (ref) | - | |
| Elementary School (Grades 1-4) | 0.992 | 0.977,1.008 | 0.750*** | 0.721,0.780 | |
| Elementary School (Grades 5+) | 0.865*** | 0.851,0.880 | 0.658*** | 0.628,0.689 | |
| High-School | 0.866*** | 0.852,0.880 | 0.549*** | 0.524,0.575 | |
| Higher Education | 0.789*** | 0.771,0.808 | 0.497*** | 0.458,0.539 | |
| Missing | 0.518*** | 0.507,0.530 | 0.492*** | 0.459,0.526 | |
| Hospital admission |  |  |  |  | |
| No | 1 (ref) | - | - |  | |
| Yes | 2.521*** | 2.489,2.553 | - |  | |
| Multimorbidity |  |  |  |  | |
| No | - |  | 1 (ref) | - | |
| Yes | - |  | 1.396*** | 1.354,1.438 | |
|  |  |  |  |  | |
| N (individuals) | 1722477 |  | 1722477 |  | |

Results from logistic regression models; PHC - Primary healthcare; AOR – Adjusted Odds Ratio. Robust standard errors used. *p<0.05; **p<0.01; *** p<0.001.

**Poisson regression results on hospitalisation rates for PHC users only**

|  | **Hospitalisation** | |  |
| --- | --- | --- | --- |
|  | **ARR** | **95% CI** | |
| Sex |  |  | |
| Male | 1 (ref) | - | |
| Female | 0.670*** | [0.659,0.680] | |
| Race |  |  | |
| White | 1 (ref) | - | |
| Black | 0.968** | [0.945,0.991] | |
| Asian | 1.231* | [1.038,1.458] | |
| Pardo (Mixed) | 0.964*** | [0.948,0.979] | |
| Indigenous | 0.886 | [0.732,1.072] | |
| Age group |  |  | |
| 0-4 years | 1 (ref) | - | |
| 0-9 years | 1.166*** | [1.124,1.210] | |
| 10-14 years | 0.807*** | [0.767,0.850] | |
| 15-19 years | 0.687*** | [0.651,0.724] | |
| 20-24 years | 0.688*** | [0.652,0.726] | |
| 25-29 years | 0.889*** | [0.839,0.942] | |
| 30-34 years | 1.021 | [0.963,1.082] | |
| 35-39 years | 1.116*** | [1.057,1.178] | |
| 40-44 years | 1.181*** | [1.120,1.246] | |
| 45-49 years | 1.228*** | [1.168,1.292] | |
| 50-54 years | 1.328*** | [1.258,1.402] | |
| 55-59 years | 1.369*** | [1.304,1.439] | |
| 60-64 years | 1.451*** | [1.386,1.519] | |
| 65-69 years | 1.562*** | [1.494,1.633] | |
| 70+ years | 1.798*** | [1.727,1.872] | |
| Bolsa Família recipient |  |  | |
| No | 1 (ref) | - | |
| Yes | 1.258*** | [1.234,1.283] | |
| Private health insurance |  |  | |
| No | 1 (ref) | - | |
| Yes | 0.651*** | [0.629,0.674] | |
| Highest education |  |  | |
| None/Pre-school/Literacy class | 1 (ref) | - | |
| Elementary School (Grades 1-4) | 0.846*** | [0.821,0.872] | |
| Elementary School (Grades 5+) | 0.876*** | [0.847,0.907] | |
| High-School | 0.792*** | [0.766,0.820] | |
| Higher Education | 0.724*** | [0.687,0.762] | |
| Missing | 0.922*** | [0.883,0.963] | |
| Multimorbidity |  |  | |
| No | 1 (ref) | - | |
| Yes | 2.735*** | [2.680,2.791] | |
|  |  |  | |
| N (individuals) | 1722477 |  | |

Results from poisson regression models; PHC - Primary healthcare; ARR – Adjusted Rate Ratio. Robust standard errors used. *p<0.05; **p<0.01; *** p<0.001.
