# Supplementary material for: Racial and socioeconomic disparities in multimorbidity and associated healthcare utilisation and outcomes in Brazil: a cross-sectional analysis of three million individuals
Source: BMC Public Health. 2021 Jul 1;21:1287. doi: 10.1186/s12889-021-11328-0 (PMC8252284; doi:10.1186/s12889-021-11328-0)
Supplement: Supplementary file 10 — Additional file 10. Sensitivity analysis with results from multiple imputation. [file 12889_2021_11328_MOESM10_ESM.docx]

**Additional File 10 – Sensitivity analysis with results from multiple imputation**

**Logistic regression results on likelihood of multimorbidity (main results and multiple imputation of missing education)**

|  | **Main results** | | **with multiple imputation** | |  |
| --- | --- | --- | --- | --- | --- |
|  | **AOR** | **95% CI** | **AOR** | **95% CI** | |
| Sex |  |  |  |  | |
| Male | 1 (ref) | - | 1 (ref) | - | |
| Female | 1.394*** | 1.382,1.407 | 1.398*** | 1.386,1.411 | |
| Race |  |  |  |  | |
| White | 1 (ref) | - | 1 (ref) | - | |
| Black | 1.045*** | 1.032,1.059 | 1.045*** | 1.032,1.059 | |
| Asian | 0.894*** | 0.845,0.946 | 0.892*** | 0.843,0.944 | |
| Pardo (Mixed) | 0.941*** | 0.933,0.950 | 0.939*** | 0.930,0.947 | |
| Indigenous | 0.815** | 0.715,0.929 | 0.823** | 0.722,0.938 | |
| Age group |  |  |  |  | |
| 0-4 years | 1 (ref) | - | 1 (ref) | - | |
| 0-9 years | 1.625*** | 1.533,1.722 | 1.610*** | 1.519,1.706 | |
| 10-14 years | 2.016*** | 1.900,2.138 | 1.991*** | 1.877,2.111 | |
| 15-19 years | 2.455*** | 2.320,2.597 | 2.412*** | 2.280,2.551 | |
| 20-24 years | 3.611*** | 3.423,3.810 | 3.503*** | 3.320,3.695 | |
| 25-29 years | 5.476*** | 5.198,5.768 | 5.291*** | 5.023,5.573 | |
| 30-34 years | 8.701*** | 8.275,9.148 | 8.401*** | 7.991,8.832 | |
| 35-39 years | 13.204*** | 12.573,13.866 | 12.771*** | 12.161,13.410 | |
| 40-44 years | 20.153*** | 19.202,21.152 | 19.536*** | 18.615,20.503 | |
| 45-49 years | 29.684*** | 28.297,31.139 | 28.773*** | 27.429,30.182 | |
| 50-54 years | 43.308*** | 41.301,45.412 | 41.943*** | 40.001,43.979 | |
| 55-59 years | 58.215*** | 55.525,61.036 | 56.326*** | 53.724,59.053 | |
| 60-64 years | 76.774*** | 73.226,80.493 | 74.272*** | 70.841,77.869 | |
| 65-69 years | 94.993*** | 90.574,99.629 | 91.877*** | 87.604,96.358 | |
| 70+ years | 109.161*** | 104.204,114.353 | 105.274*** | 100.495,110.279 | |
| Bolsa Família recipient |  |  |  |  | |
| No | 1 (ref) | - | 1 (ref) | - | |
| Yes | 1.140*** | 1.126,1.153 | 1.189*** | 1.175,1.203 | |
| Private health insurance |  |  |  |  | |
| No | 1 (ref) | - | 1 (ref) | - | |
| Yes | 0.758*** | 0.744,0.771 | 0.764*** | 0.751,0.778 | |
| Highest education |  |  |  |  | |
| None/Pre-school/Literacy class | 1 (ref) | - | 1 (ref) | - | |
| Elementary School (Grades 1-4) | 0.994 | 0.978,1.009 | 0.982* | 0.966,0.997 | |
| Elementary School (Grades 5+) | 0.866*** | 0.852,0.882 | 0.869*** | 0.854,0.884 | |
| High-School | 0.868*** | 0.854,0.882 | 0.864*** | 0.850,0.878 | |
| Higher Education | 0.793*** | 0.774,0.811 | 0.794*** | 0.775,0.813 | |
| Missing | 0.521*** | 0.510,0.533 |  |  | |
| PHC user |  |  |  |  | |
| No | 1 (ref) | - | 1 (ref) | - | |
| Yes | 172.897*** | 164.783,181.411 | 236.150*** | 225.239,247.589 | |
| Hospital admission |  |  |  |  | |
| No | 1 (ref) | - | 1 (ref) | - | |
| Yes | 2.671*** | 2.637,2.706 | 2.660*** | 2.626,2.695 | |
|  |  |  |  |  | |
| N (individuals) | 3027335 |  | 3027335 |  | |

Results from logistic regression models; PHC - Primary healthcare; AOR – Adjusted Odds Ratio. Robust standard errors used. *p<0.05; **p<0.01; *** p<0.001.

**Logistic regression results on likelihood of death (main results and multiple imputation of missing education)**

|  | ARR | 95% CI | ARR | 95% CI |
| --- | --- | --- | --- | --- |
| Sex |  |  |  |  |
| Male | 1 (ref) | - | 1 (ref) | - |
| Female | 0.581*** | 0.569,0.593 | 0.577*** | 0.565,0.589 |
| Race |  |  |  |  |
| White | 1 (ref) | - | 1 (ref) | - |
| Black | 1.166*** | 1.130,1.203 | 1.140*** | 1.104,1.176 |
| Asian (*Amarelo*) | 1.231** | 1.087,1.395 | 1.237*** | 1.091,1.401 |
| Pardo (Mixed) | 0.971* | 0.950,0.993 | 0.959*** | 0.937,0.980 |
| Indigenous | 1.106 | 0.832,1.469 | 1.08 | 0.812,1.436 |
| Age group |  |  |  |  |
| 0-4 years | 1 (ref) | - | 1 (ref) | - |
| 0-9 years | 0.243*** | 0.192,0.307 | 0.258*** | 0.204,0.326 |
| 10-14 years | 0.266*** | 0.209,0.338 | 0.281*** | 0.221,0.358 |
| 15-19 years | 1.074 | 0.923,1.250 | 1.167* | 1.003,1.358 |
| 20-24 years | 1.753*** | 1.529,2.011 | 2.148*** | 1.875,2.460 |
| 25-29 years | 1.749*** | 1.523,2.008 | 2.212*** | 1.929,2.536 |
| 30-34 years | 1.820*** | 1.587,2.087 | 2.345*** | 2.048,2.686 |
| 35-39 years | 2.306*** | 2.021,2.630 | 2.978*** | 2.613,3.395 |
| 40-44 years | 3.123*** | 2.746,3.551 | 4.014*** | 3.533,4.561 |
| 45-49 years | 4.069*** | 3.589,4.614 | 5.216*** | 4.603,5.911 |
| 50-54 years | 6.347*** | 5.624,7.162 | 8.129*** | 7.207,9.168 |
| 55-59 years | 9.974*** | 8.862,11.225 | 12.737*** | 11.322,14.328 |
| 60-64 years | 14.854*** | 13.216,16.696 | 18.915*** | 16.837,21.250 |
| 65-69 years | 21.662*** | 19.282,24.335 | 27.606*** | 24.585,30.998 |
| 70+ years | 64.473*** | 57.591,72.178 | 81.604*** | 72.929,91.311 |
| Bolsa Familia recipient |  |  |  |  |
| No | 1 (ref) | - | 1 (ref) | - |
| Yes | 1.517*** | 1.460,1.577 | 1.246*** | 1.201,1.293 |
| Private health insurance |  |  |  |  |
| No | 1 (ref) | - | 1 (ref) | - |
| Yes | 0.820*** | 0.793,0.849 | 0.907*** | 0.877,0.938 |
| Highest education |  |  |  |  |
| None/Pre-school/Literacy class | 1 (ref) | - | 1 (ref) | - |
| Elementary School (Grades 1-4) | 0.788*** | 0.759,0.819 | 0.766*** | 0.737,0.796 |
| Elementary School (Grades 5+) | 0.731*** | 0.700,0.763 | 0.828*** | 0.797,0.861 |
| High-School | 0.593*** | 0.568,0.620 | 0.612*** | 0.588,0.637 |
| Higher Education | 0.546*** | 0.505,0.590 | 0.558*** | 0.522,0.597 |
| Missing | 1.288*** | 1.240,1.338 |  |  |
| Multimorbidity |  |  |  |  |
| No | 1 (ref) | - | 1 (ref) | - |
| Yes | 1.327*** | 1.293,1.361 | 1.052*** | 1.027,1.077 |
|  |  |  |  |  |
| N | 3027335 |  | 3027335 |  |

Results from logistic regression models; PHC - Primary healthcare; AOR – Adjusted Odds Ratio. Robust standard errors used. *p<0.05; **p<0.01; *** p<0.001.
